# Proton‐Coupled Electron Transfer on Cu_2_O/Ti_3_C_2_T_x_ MXene for Propane (C_3_H_8_) Synthesis from Electrochemical CO_2_ Reduction

**DOI:** 10.1002/advs.202405154

**Published:** 2024-08-19

**Authors:** Jun Young Kim, Won Tae Hong, Thi Kim Cuong Phu, Seong Chan Cho, Byeongkyu Kim, Unbeom Baeck, Hyung‐Suk Oh, Jai Hyun Koh, Xu Yu, Chang Hyuck Choi, Jongwook Park, Sang Uck Lee, Chan‐Hwa Chung, Jung Kyu Kim

**Affiliations:** ^1^ School of Chemical Engineering Sungkyunkwan University (SKKU) 2066, Seobu‐ro, Jangan‐gu Suwon 16419 Republic of Korea; ^2^ Clean Energy Research Center Korea Institute of Science and Technology (KIST) Hwarang‐ro 14‐gil 5, Seongbuk‐gu Seoul 02792 Republic of Korea; ^3^ School of Chemistry and Chemical Engineering Yangzhou University Yangzhou 225002 P. R. China; ^4^ Department of Chemistry Pohang University of Science and Technology (POSTECH) Pohang 37673 Republic of Korea; ^5^ Institute of Convergence Research and Education in Advanced Technology (I‐CREATE) Yonsei University Seoul 03722 Republic of Korea; ^6^ Integrated Engineering Department of Chemical Engineering Kyung Hee University Gyeonggi 17104 South Korea; ^7^ SKKU Advanced Institute of Nano Technology (SAINT) Sungkyunkwan University 2066 Seobu‐ro Suwon 16419 Republic of Korea

**Keywords:** C2‐C1 coupling, electrochemical CO2 reduction, in‐situ ATR‐FTIR, propane production, proton‐coupled electron transfer

## Abstract

Electrochemical CO_2_ reduction reaction (CO_2_RR) to produce value‐added multi‐carbon chemicals has been an appealing approach to achieving environmentally friendly carbon neutrality in recent years. Despite extensive research focusing on the use of CO_2_ to produce high‐value chemicals like high‐energy‐density hydrocarbons, there have been few reports on the production of propane (C_3_H_8_), which requires carbon chain elongation and protonation. A rationally designed 0D/2D hybrid Cu_2_O anchored‐Ti_3_C_2_T_x_ MXene catalyst (Cu_2_O/MXene) is demonstrated with efficient CO_2_RR activity in an aqueous electrolyte to produce C_3_H_8_. As a result, a significantly high Faradaic efficiency (FE) of 3.3% is achieved for the synthesis of C_3_H_8_ via the CO_2_RR with Cu_2_O/MXene, which is ≈26 times higher than that of Cu/MXene prepared by the same hydrothermal process without NH_4_OH solution. Based on in‐situ attenuated total reflection‐Fourier transform infrared spectroscopy (ATR‐FTIR) and density functional theory (DFT) calculations, it is proposed that the significant electrocatalytic conversion originated from the synergistic behavior of the Cu_2_O nanoparticles, which bound the *C_2_ intermediates, and the MXene that bound the *CO coupling to the C_3_ intermediate. The results disclose that the rationally designed MXene‐based hybrid catalyst facilitates multi‐carbon coupling as well as protonation, thereby manipulating the CO_2_RR pathway.

## Graphical abstract

A rational design of Cu_2_O/Ti_3_C_2_T_x_ MXene catalyst presents an excellent activity for saturated hydrocarbon C_3_H_8_ from electrochemical CO_2_ reduction, providing ^*^C_2_‐^*^C_1_ coupling sites and facilitating further protonation. Based on *in‐situ* attenuated total reflection‐Fourier transform infrared spectroscopy and density functional theory calculations, results offer insights into green electrochemical carbon utilization for achieving carbon neutrality as well as lowering global warming potential.

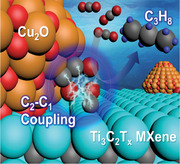

## 1 Introduction

With the continuously increasing demand to reduce CO_2_ emissions, the conversion of CO_2_ into value‐added hydrocarbon products has gained significant attention.^[^ ^1^ ^]^ From an energy perspective, utilization of CO_2_ to produce high‐value chemicals, particularly those with high energy density, such as hydrocarbons with long carbon chains or saturated (i.e., hydrogen‐rich) hydrocarbons, is desirable. In particular, propane (C_3_H_8_) has a high specific energy (50.4 MJ kg^−1^) with a remarkably low global warming potential (< 1) compared to other CO_2_‐driven products including CO, CH_4_, and C_2_H_4_, thereby considered a desirable renewable energy source to reduce the impact on carbon footprint with versatile applications.^[^ ^2^ ^]^ Among the various technologies to utilize CO_2_ to produce C_3_H_8_, the electrochemical conversion of CO_2_ can be effective since the electrochemical CO_2_ reduction reaction (CO_2_RR) operates under environmentally benign mild conditions, using renewable electricity. However, the electrochemical CO_2_RR to C_3_H_8_ in an aqueous electrolyte is challenging because the reaction involves several steps, including carbon chain elongation and protonation with 20 electrons. Thus, modulating the electrochemical catalysts to promote the protonation of longer‐chain carbons is critical to produce C_3_H_8_. To the best of our knowledge, few studies report a notable Faradaic efficiency for C_3_H_8_ of the electrochemical CO_2_RR in an aqueous electrolyte, but most of the research achieved a modest Faraday efficiency (FE) of ≈1%, without the aid of other ionomers or carbon sources.^[^ ^3^ ^]^

A recent study with imidazolium‐functionalized Mo_3_P coated with the ionomer (ImF‐Mo_3_P) showed FE of 91% C_3_H_8_, marking a significant advance over previous reports that typically showed FE less than 1% or even trace amounts.^[^ ^4^ ^]^ However, the carbon sources, i.e., carbon functional groups, namely imidazolium moieties on the catalyst, as well as the coated ionomers, are participating in the CO_2_RR.^[^ ^5^ ^]^ These carbon sources inhibit the identification of the reaction mechanism to find the crucial *C_2_ and *C_3_ intermediates for C_3_H_8_. Especially, *in‐situ* Raman data pinpointing appropriate CO_2_RR intermediates other than *CO to support the proposed reaction mechanism from CO_2_ to C_3_H_8_ is required. Given that the *CO formation is an initial step of CO_2_RR, peaks for *CO alone cannot directly substantiate the C_3_H_8_ production. Thus, a rational strategy by facilitating multi‐carbon coupling and protonation supported by substantial experimental evidence to represent the reaction mechanism is required.

Cu‐based catalyst is generally considered to produce multiple hydrocarbons of various chain lengths from CO_2_.^[^ ^6^ ^]^ Some studies on Cu‐based catalysts reported a wide selectivity for C_3_ products including n‐propanol, acetone, and hydroxyacetone,^[^ ^7^ ^]^ albeit the FE of C_3_H_8_ is generally less than 0.3%, or even a traceable amount, indicating a lack of protonation.^[^ ^8^ ^]^ To increase the selectivity toward multi‐carbon, Zhang et al. reported that not only the electron transfer for CO dimerization at catalytic active sites but also several protonation steps are necessary.^[^ ^9^ ^]^

Ti_3_C_2_T_x_ MXene with 2D layered structure has received considerable attention due to its catalytic performance in the electrochemical CO_2_RR with excellent chemical durability and abundant adsorption sites with tunable functional groups on the surface.^[^ ^10^ ^]^ Especially, in an aqueous electrolyte, the naturally modulated O‐ and OH‐ functional group of MXene can share H with CO_2_RR intermediates, enabling proton‐coupled electron transfer (PCET) for the protonation of hydrocarbon products, thus increasing the energy density of CO_2_ reduction products.^[^ ^11^ ^]^ Besides, modulating the MXene functional groups with O‐ or OH‐ can result in high chemical reactivity and an increase in the number of active sites for catalytic reactions in an aqueous electrolyte.^[^ ^12^ ^]^ Thus, the composite of Cu_2_O and MXene can be useful for hydrocarbon generation with long‐term stability, given that the modulated MXene surfaces and oxygen‐derived Cu offer stable adsorption sites for CO_2_ molecules, further promoting C‐C coupling and effective protonation of longer‐chain hydrocarbons.^[^ ^11^, ^13^ ^]^

In this study, we designed a rational hybrid structure of Cu_2_O incorporated into MXene (Cu_2_O/MXene) for the efficient conversion of CO_2_ into multi‐carbon products, especially C_3_H_8_ which requires C_3_ coupling and sufficient protonation. To understand the synergistic effect of the Cu_2_O and MXene with the mechanism of CO_2_ conversion to C_3_H_8_, Cu_2_O/MXene was characterized by X‐ray photoelectron spectroscopy (XPS), Fourier‐transform infrared spectroscopy (FT‐IR), Auger electron spectroscopy (AES) and X‐ray absorption spectroscopy (XAS). Notably, attenuated total reflection‐Fourier transform infrared spectroscopy (ATR‐FTIR) revealed that the interfacial effect between Cu_2_O and MXene was significant for *C_2_–*C_1_ coupling. Specifically, Cu_2_O strongly bound and preserved *C_2_ intermediates. In contrast, the MXene bound to the sole *C_1_ site and provided sufficient protons to the CO_2_RR intermediates. As a result, Cu_2_O/MXene reveals an efficient C_3_H_8_ production with a FE of 3.3% at −1.3 V versus reversible hydrogen electrode (RHE) in CO_2_ saturated 0.1 M KHCO_3_, without the aid of carbon sources. We envision that our strategy of catalyst design combined with Cu_2_O and MXene, which could tune the selectivity toward multi‐carbon products and proton‐coupled electron transfer, respectively, is able to produce saturated hydrocarbon products with high energy density, such as C_3_H_8_.

## 2 Results

### 2.1 Characterizations for Cu_2_O/MXene

Cu_2_O/MXene was fabricated via a facile hydrothermal process. First, Ti_3_C_2_T_x_ MXene nanosheets were prepared by a selective Al etching method, starting from Ti_3_AlC_2_ MAX powder with further delamination, following the previous study with some modifications.^[^ ^14^ ^]^ Cu nanoparticles (CuNPs) were prepared separately by the hot‐injection method. After individually fabricating MXene and CuNPs, the mixture solution of MXene and CuNPs was transferred to a Teflon liner, with 4 mmol NH_4_OH solution added to adjust pH ≈10, and autoclaved for 30 min at 70 °C. During the synthesis, some of the functional groups of the MXene nanosheets were modulated to hydroxyl (‐OH) moieties in the presence of an NH_4_OH solution. In addition, the NH_4_OH solution induced partial oxidation of CuNPs to form Cu_2_O and further consolidated Cu_2_O on the MXene. The as‐prepared samples were then freeze‐dried to prevent further oxidation and restacking. To compare the CO_2_RR activity, Cu/MXene was synthesized in the same manner as Cu_2_O/MXene without the use of an NH_4_OH solution, so the CuNPs were not significantly oxidized when anchored to MXene during the synthesis.

**Figure 1:**
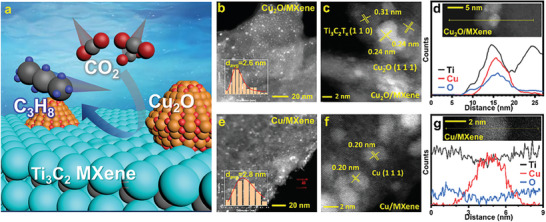
a) Schematic illustration of CO_2_RR with Cu_2_O/MXene. TEM, HRTEM, and EDS line profile mapping for b–d) Cu_2_O/MXene and e–g) Cu/MXene. b) and e) TEM images of Cu_2_O/MXene and Cu/MXene. c) and f) HRTEM for Cu_2_O/MXene and Cu/MXene. d) and g) EDS line profile mapping Ti, Cu, and O for Cu_2_O/MXene and Cu/MXene.

Transmission electron microscopy (TEM) was used to examine the structural morphology of Cu_2_O/MXene (**Figure** **1**). As shown in Figure 1a‐b, the Cu NPs adhered to the MXene nanosheets. As shown in Figure 1b, the CuNPs were distributed on the surface of MXene with 2.6 nm of average diameter, showing a typical log‐normal distribution. The high‐resolution TEM (HRTEM) image in Figure 1c shows that the CuNPs in Cu_2_O/MXene were crystalline structures with lattice fringes of 0.24 nm for Cu_2_O (111). This indicates the partial oxidation of CuNPs during the hydrothermal process in the presence of NH_4_OH solution to form Cu_2_O. However, no morphological variance was observed between MXene and NH_4_OH‐treated MXene (AT‐MXene), as shown in Figure S1 (Supporting Information). In addition, a clear interface was observed between the CuNPs and MXene. EDS line profile scanning was conducted to assign Ti (black), Cu (red), and O (blue) (Figure 1d,g), where a clear interface between the CuNPs and MXene was observed. The elemental O distribution was higher in the presence of Cu, indicating the incorporation of CuNPs as Cu_2_O phase into MXene.

As for the Cu/MXene, which was synthesized without NH_4_OH treatment, the average diameter of the CuNPs on Cu/MXene was 2.8 nm with a narrower size distribution compared to Cu_2_O/MXene (Figure 1e). In addition, the CuNPs in Cu/MXene, which predominantly presents a lattice fringe with 0.20 nm for Cu (111), are metallic Cu (Figure 1f). In Figure 1g, metallic CuNPs are observed with no change in the elemental O distribution, whereas an increase in the Ti content is observed in Cu/MXene. Thus, the presence of NH_4_OH during the synthesis induced the incorporation of CuNPs being transformed to Cu_2_O on the surface of the MXene nanosheets.

**Figure 2:**
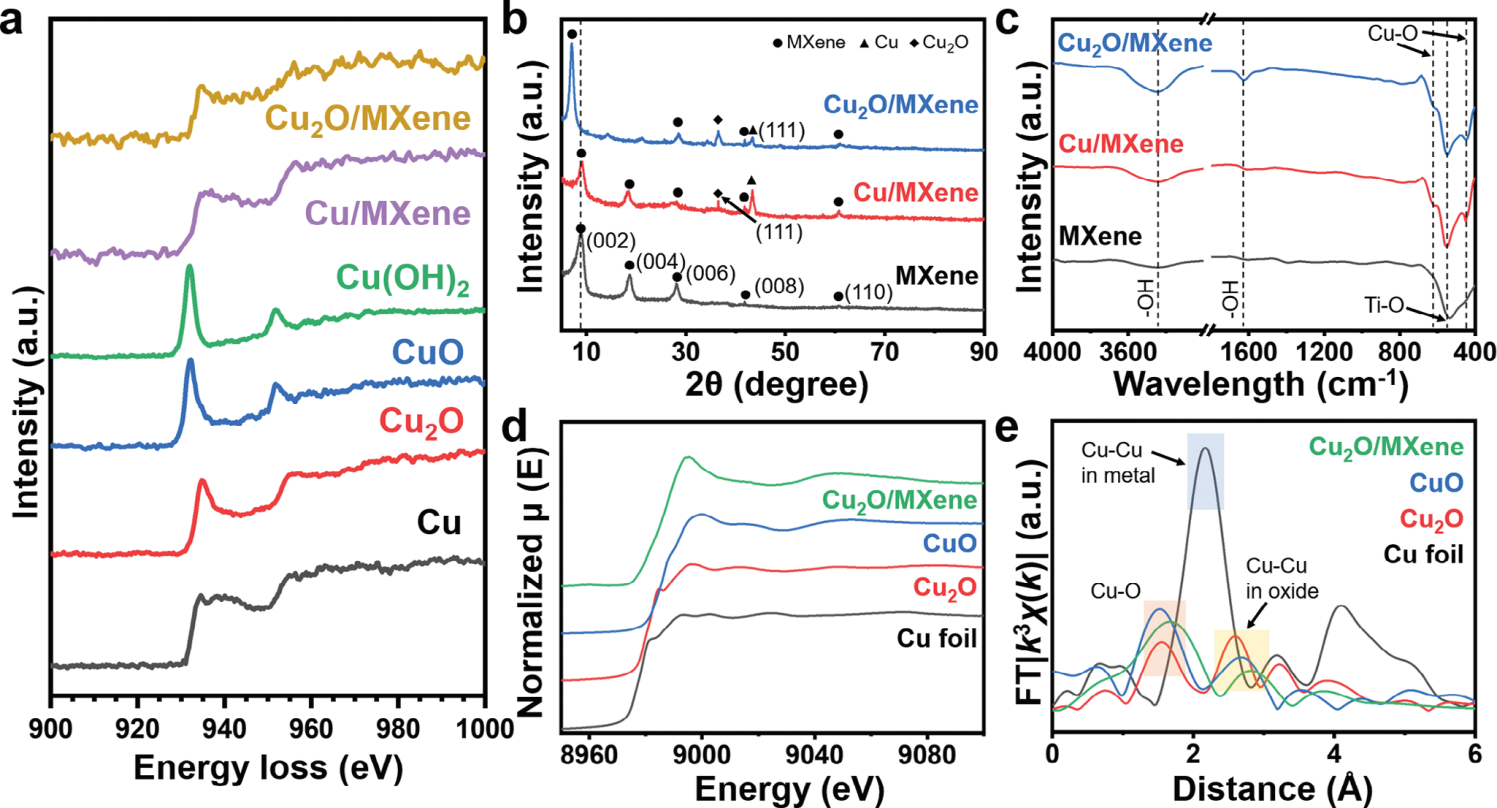
a) EELS spectra of Cu/MXene and Cu_2_O/MXene. b) XRD and c) FT‐IR spectra of MXene, Cu/MXene and Cu_2_O/MXene. d) XANES and e) FT‐EXAFS data for Cu_2_O/MXene.

Following up on the TEM results, electron energy‐loss spectrometry (EELS) was performed to determine the valence states of the Cu species in Cu_2_O/MXene and Cu/MXene (**Figure** **2**a). From the EELS spectra, the slope for Cu_2_O/MXene in the range of 930–940 eV was between those of Cu and Cu_2_O, but close to that of Cu_2_O. Therefore, it was difficult to distinguish the valence state of Cu in Cu/MXene from that in Cu_2_O/MXene. These results may imply that the valence state of Cu in both Cu_2_O/MXene and Cu/MXene was a combination of metallic Cu and Cu^+^. Additionally, by comparing the EELS spectra of Cu_2_O/MXene and Cu/MXene in the range of 400–750 eV, representing MXene, we found a low residual F intensity with a higher O intensity in Cu_2_O/MXene (Figure S2, Supporting Information). This indicates that the NH_4_OH treatment induced not only the partial oxidation of CuNPs but also the successful functional group modulation of MXene in Cu_2_O/MXene during the hydrothermal process.

The crystal and phase structures of Cu_2_O/MXene were characterized by XRD (Figure 2b). The two distinct main peaks (i.e., (002) and (004)) represent MXene, which is consistent with the results of previous studies.^[^ ^15^ ^]^ The (002) peak at 8.87° is a typical peak for the Ti_3_C_2_T_x_ MXene, indicating a c‐lattice parameter (c‐LP, ≈ 19.9 Å).^[^ ^16^ ^]^ This (002) peak did not shift in Cu/MXene. However, the (002) peak shifted toward a lower value (7.06°) in Cu_2_O/MXene with c‐LP to 25.0 Å when Cu_2_O was anchored to the MXene. This result may imply that the CuNPs were anchored to the functional group of MXene as Cu_2_O phase, affecting the lattice structure and eventually increasing the interlayer distance of MXene.^[^ ^17^ ^]^ In Cu_2_O/MXene, the composition of Cu_2_O (111) was higher than the Cu (111) (JCPDS 77–0199 and JCPDS 04–0836, respectively). The ratio from the quantitative phase analysis by Rietveld refinement for Cu:Cu_2_O was 0.17:0.83, indicating a high fraction of Cu_2_O. A sharp peak with low intensity for Cu_2_O (111) was also observed for Cu/MXene. Unlike Cu_2_O/MXene, the ratio of Cu:Cu_2_O in Cu/MXene was 0.91:0.09, indicating that most of the CuNPs were composed of Cu^0^. Thus, NH_4_OH treatment affected the oxidation state of Cu in Cu_2_O/MXene.

FT‐IR was conducted to determine the chemical bonds and functional groups on the surface of Cu_2_O/MXene. As shown in Figure 2c, the peak at 540 cm^−1^ is attributed to the vibration of the Ti‐O bond from the MXene.^[^ ^18^ ^]^ Cu‐O stretching vibration peaks are also observed at 425 cm^−1^ and 620 cm^−1^,^[^ ^19^ ^]^ presumably the CuNPs were hybridized onto the ‐O or ‐OH functional group of MXene forming Cu_2_O. The broad absorption band with a peak at 3445 cm^−1^ corresponds to the ‐OH stretching vibration of the water molecules in the MXene interlayer.^[^ ^20^ ^]^ For the Cu_2_O/MXene where NH_4_OH treatment was applied, the FT‐IR spectra showed a slight peak at 1625 cm^−1^ which is attributed to the OH groups.^[^ ^21^ ^]^ Thus, this result suggests that the NH_4_OH treatment modulated the functional group of the MXene to the ‐OH.

The coordination environment of the CuNPs in Cu_2_O/MXene was further characterized by synchrotron‐based X‐ray absorption near‐edge structure (XANES) and extended X‐ray absorption fine structure (EXAFS). As shown in Figure 2d, the Cu absorption edge of Cu_2_O/MXene resided between those of commercial Cu and Cu_2_O. Especially, the Cu k‐edge for Cu_2_O/MXene was in between those of Cu_2_O and CuO but close to that of Cu_2_O, suggesting that the average oxidation state of Cu is likely to be higher than that of Cu^+^ but lower than that of Cu^2+^. This valence state also reflected the successful hybridization of Cu_2_O and MXene, where Cu_2_O was anchored to MXene. The Fourier transform (FT)‐EXAFS spectra of Cu_2_O/MXene and the references (Figure 2e) showed the intensity of the Cu‐O peak at ≈1.6 Å, which was intermediate between the intensities found in Cu_2_O and CuO. The increase in Cu‐O distance on Cu_2_O/MXene also shows evidence of Cu‐O‐Ti bonding by hybridization, as the length of the Cu‐O bond from the Cu‐O‐Cu is shorter than the Cu‐O‐Ti bond length.^[^ ^22^ ^]^ The Cu‐Cu peak from the oxide (≈2.8 Å) is distinct in Cu_2_O/MXene which is associated with the scattering path of the second Ti shell (i.e., Ti L‐shell).^[^ ^22^ ^]^

**Figure 3:**
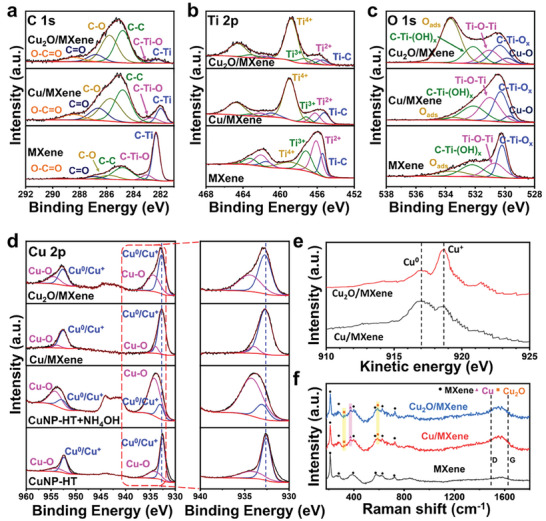
XPS spectra of a) C 1s, b) O 1s, and c) Ti 2p of MXene, Cu/MXene and Cu_2_O/MXene. d) Cu 2p of Cu/MXene and Cu_2_O/MXene displayed with a magnified view of XPS Cu 2p_3/2_ comparing with CuNP prepared with hydrothermal process in the presence and absence of NH_4_OH treatment. e) Cu LMM AES spectra of Cu/MXene and Cu_2_O/MXene. f) Raman spectra of MXene, Cu/MXene, and Cu_2_O/MXene.

XPS was further performed to elucidate the chemical structure of Cu_2_O anchored to MXene from Cu_2_O/MXene, compared with MXene and Cu/MXene (**Figure** **3**). In the XPS high‐resolution spectra of C 1s (Figure 3a), the binding energy peak at 284.8 eV was assigned to the adventitious carbon of C‐C.^[^ ^20^ ^]^ Furthermore, the binding energy of C‐Ti in Cu_2_O/MXene shifted to a lower value (282.3 eV) than that of bare MXene (282.6 eV), indicating that Cu_2_O transferred electrons to MXene. The oxygen contents with carbon (C‐O, C = O, and O‐C = O) peaks may result from MXene oxidation and carbon networks.^[^ ^23^ ^]^ In the XPS Ti 2p spectra (Figure 3b), the binding energies of Ti‐C, Ti^2+^, Ti^3+^, and Ti^4+^ for MXene are 455.4, 456.1, 457.3, and 458.8 eV, respectively. Specifically, the Ti^2+^, Ti^3+^, and Ti^4+^ peaks are attributed to Ti‐X, Ti‐O, and Ti‐O_x_ (including Ti‐(OH)_x_), respectively.^[^ ^24^ ^]^ When CuNPs are hybridized onto the MXene (Cu/MXene and Cu_2_O/MXene), the CuNPs are consolidated at the oxygen‐rich functional group of MXene. The evidence of anchoring CuNPs on MXene is further characterized by the O 1s XPS. The O 1s region of the MXene was deconvoluted by components corresponding to C‐Ti‐O_x_, Ti‐O‐Ti, C‐Ti‐(OH)_x_, O_ads_ at BE = 530.5, 531.2, 532.0, and 533.2 eV, respectively (Figure 3c).^[^ ^25^ ^]^ O_ads_ is the surface adsorbed oxygen species in MXene, and the peak at BE = 533.2 eV corresponds to adsorbed water.^[^ ^26^ ^]^ The peak for the adsorbed water was significantly increased when Cu_2_O was applied to the MXene, affecting the hydrophilicity. In addition, compared to the MXene, Cu/MXene and Cu_2_O/MXene display a positive shift of C‐Ti‐O_x_ and Ti‐O‐Ti, demonstrating that the CuNPs were anchored onto the MXene terminals. Notably, the N 1s peak in AT‐MXene or Cu_2_O/MXene was not observed, indicating that the NH_4_OH treatment affected the functional group modulation of MXene to the ‐OH group and did not participate in the N‐doped or amine groups (Figure S7, Supporting Information). Besides, as MXene was fabricated and stored as a solution, the MXene already has some ‐OH functional groups, even without NH_4_OH treatment. Thus, the NH_4_OH treatment slightly increased the number of ‐OH functional groups. The result is in accordance with the previous research by Gogotsi's group, which modulated the MXene functional group by the NH_4_OH solution to the ‐OH.^[^ ^27^ ^]^

To determine the valence state of Cu, the Cu 2p XPS for Cu_2_O/MXene was compared with that of Cu/MXene, as well as CuNPs with the hydrothermal process (CuNP‐HT) in the absence and presence of NH_4_OH treatment (Figure 3d). The Cu^0^/Cu^+^ and Cu^2+^ peaks of Cu 2p_3/2_ were observed at 933.0 and 934.2 eV. Without MXene, the NH_4_OH solution easily oxidized the surface of CuNP to form Cu_2_O or even further oxidized to CuO. Thus, a significant increase in the Cu^2+^ peak was observed compared with CuNP‐HT and Cu_2_O (CuNP‐HT+NH_4_OH). The typical Cu^2+^ satellites at 940–945 eV and 960–965 eV also confirmed the further oxidation of Cu to CuO with NH_4_OH treatment in the absence of MXene, in accordance with the XRD data (Figure S3, Supporting Information).^[^ ^28^ ^]^ In the presence of MXene, although the Cu^0^/Cu^+^ peak was shifted to a higher binding energy, NH_4_OH treatment cannot fully oxidize Cu to Cu^2+^ since NH_4_OH also affects the partial substitution of the functional group of MXene to ‐OH. Cu LMM Auger electron spectra (AES) were tested to further carefully distinguish the oxidation state of the CuNPs decorated on MXene (Figure 3e). Comparing the intensities of Cu^0^ and Cu^+^, Cu^+^ was dominant in Cu_2_O/MXene, whereas Cu^0^ was dominant in Cu/MXene.

The chemical structure of Cu_2_O/MXene was revealed using Raman spectroscopy (Figure 3f). Signals representing the vibrations of Ti and C atoms were observed in MXene, Cu/MXene, and Cu_2_O/MXene (i.e., peaks at 201, 274, 386, 582, 626, and 726 cm^−1^).^[^ ^10^ ^]^ The peak located at 201 cm^−1^ is attributed to the out‐of‐plane vibrations of Ti atoms, whereas the peaks at 274 and 386 cm^−1^ are ascribed to the in‐plane vibrations of the surface groups attached to the Ti atoms.^[^ ^29^ ^]^ The two peaks at 1373 cm^−1^ and 1576 cm^−1^ represent the D band and G band, respectively, where the former D‐band is generally related to the disordered graphite formed by defects in carbon‐based materials, whereas the latter G‐band is ascribed to the stacking of the graphite hexagonal network plane.^[^ ^10^, ^15^ ^]^ For Cu/MXene and Cu_2_O/MXene, both Cu and Cu_2_O were observed (376 cm^−1^ for Cu, and 322 and 590 cm^−1^ for Cu_2_O, respectively).^[^ ^30^ ^]^ Thus, Cu could be slightly oxidized when anchored to MXene, even when the NH_4_OH treatment was not applied to Cu/MXene.

### 2.2 Electrochemical Analysis for CO_2_ Reduction

**Figure 4:**
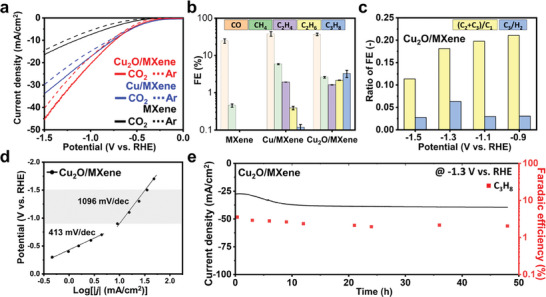
Electrochemical tests of Cu_2_O/MXene. a) LSV curves of Cu/MXene and Cu_2_O/MXene saturated with CO_2_ (straight lines) and Ar (dashed lines). Applied potential is corrected with iR compensation, and ECSA is considered for current density. b) Faradaic efficiency on each carbonaceous CO_2_RR product at −1.3 V versus RHE. Error bars represent the standard deviation in FE calculated after three tests for repeatability. The other non‐carbon product is listed in Table S2 (Supporting Information). c) Relative ratio of the FE with Cu_2_O/MXene. d) Tafel slope with Cu_2_O/MXene. The intermediate overpotential region where significant C_3_ production was detected is highlighted in grey. e) Stability tests of Cu_2_O/MXene at −1.3 V versus RHE.

Linear sweep voltammetry (LSV) curves in Ar and CO_2_ saturated electrolyte were used to evaluate the CO_2_RR performance of Cu_2_O/MXene, and then the results were compared with those of MXene and Cu/MXene (**Figure** **4**a). MXene, Cu/MXene, and Cu_2_O/MXene showed activity on CO_2_RR. Anchoring Cu_2_O on MXene further increased the activities of both CO_2_RR and hydrogen evolution reaction (HER), according to the results obtained with CO_2_ and Ar saturation. The current density of Cu_2_O/MXene in CO_2_ purged electrolyte was higher than that in the Ar, demonstrating its good electrocatalytic activity for CO_2_ reduction. In addition, the CO_2_ saturated LSV curves for all samples exhibited more positive onset potentials than the Ar‐saturated ones, suggesting that their activities on CO_2_ participated in the reaction. Note that these current density data can be enhanced with different cell configuration such as a membrane electrode assembly (MEA) cell. The electrochemical impedance spectroscopy (EIS) was conducted to investigate the kinetic behaviors of the electrocatalyst for CO_2_RR in CO_2_‐saturated 0.1 M KHCO_3_ (Figure S9, Supporting Information). For the Cu NPs showed low charge transfer resistance reflecting efficient CO_2_RR activity in high frequency region but extremely large diffusion resistance in low frequency region. In cases of MXene and AT‐MXene, both exhibited large semi‐circles which indicate insufficient CO_2_RR, which are coherent to the product analysis. The Cu/MXene showed smaller semi‐circle than those of MXene and AT‐MXene by enhanced charge transfer due to the Cu anchoring. Especially, the Cu_2_O/MXene displayed the lowest charge transfer resistance among the MXene‐based electrocatalysts reflecting superior activity for CO_2_RR. From the CO_2_RR, products with CO, CH_4_, C_2_H_4_, C_2_H_6_, and C_3_H_8_ were observed. The FEs of the CO_2_RR products at the applied potentials for MXene, AT‐MXene, Cu/MXene, and Cu_2_O/MXene are shown in Figure S13 (Supporting Information). As shown in Figure S14 (Supporting Information), the GC‐FID chromatograms clearly confirm that the gaseous product is comprised with CH_4_, C_2_H_4_, and C_3_H_8_, not including C_3_H_6_. The FE of the CO_2_RR products on Cu_2_O/MXene at −1.3 V versus RHE was compared with that of Cu/MXene in Figure 4b, where the highest FE_C3H8_ was observed. Cu_2_O/MXene was the most active electrocatalyst, affording 3.3% of FE_C3H8_ at −1.3 V versus RHE as the optimum potential, suggesting effective electron and proton transfer during CO_2_ reduction. These results were comparable to those obtained for Cu/MXene with ≈0.1% FE_C3H8_. Although MXene also exhibited activity in CO_2_, the products of the CO_2_RR were C_1_ gases composed of CO and CH_4_. Especially, as the major products of Cu or Cu_2_O are C_1_ and C_2_ products,^[^ ^31^ ^]^ the presence of MXene in the composite may affect the C_3_ intermediate production toward C_3_H_8_. We also compared the experimental C_2_ & C_3_ to C_1_ ratio with −0.9 to −1.5 V versus RHE, as shown in Figure 4c. When the applied potential increased, the (C_2_+C_3_)/C_1_ ratio decreased, indicating that the time for carbon coupling became insufficient as the HER is significantly affected by the higher applied potential, which is the competitive reaction of CO_2_RR. Additionally, the FE of both CO and CH_4_ decreased with increasing applied potential (Figure S13, Supporting Information), indicating that the HER favored over the CO_2_RR.^[^ ^7a^ ^]^ An interesting result on the ratio of C_3_ to H_2_ was found at −1.3 V versus RHE, showing the highest value. In general, the C_3_/H_2_ ratio is low as *H adsorption onto the catalyst during the HER requires less energy than the protonation of the hydrocarbon products.^[^ ^3b^ ^]^ However, as the MXene could provide protons to the C_3_ intermediate, the highest C_3_H_8_ FE at −1.3 V versus RHE could be achieved by a proton‐coupled electron‐transfer step, while concurrently reducing the HER.^[^ ^11^ ^]^

Generally, selectivity is highly dependent on the competitive adsorption of intermediates such as *CO and *OCCO, as well as the reaction kinetic energy barrier of the rate‐determining step in the electrochemical CO_2_RR for C_3_H_8_ production.^[^ ^32^ ^]^ The Tafel plot was tested for Cu_2_O/MXene and Cu/MXene, highlighting the CO_2_RR intermediate overpotential region (−0.9 to −1.5 V versus RHE) where significant C_3_ products were observed (Figure 4d; Figure S15, Supporting Information). The Tafel slope of the Cu/MXene at −0.9 to −1.5 V versus RHE was 957 mV dec^−1^, lower than that of Cu_2_O/MXene (1096 mV dec^−1^). However, the C_2_ and C_3_ production for Cu/MXene was much lower than that for Cu_2_O/MXene (Figure 4b). These results indicate sluggish reaction kinetics for the C_2_ and C_3_ hydrocarbon production which require complex multistep reactions including carbon coupling and protonation.^[^ ^33^ ^]^ Thus, Cu_2_O/MXene could effectively enforce the reaction pathway toward multi‐carbon production when sufficient time for carbon coupling and protonation was provided during electrochemical CO_2_ reduction. In addition, the long‐term stability of Cu_2_O/MXene at −1.3 V versus RHE up to 48 h was investigated as shown in Figure 4e. After the stability test, Cu_2_O NPs stably anchored onto the MXene were observed by HAADF‐STEM, and the catalyst was characterized by XRD and XPS, which showed no significant changes (Figure S17, Supporting Information).

**Figure 5:**
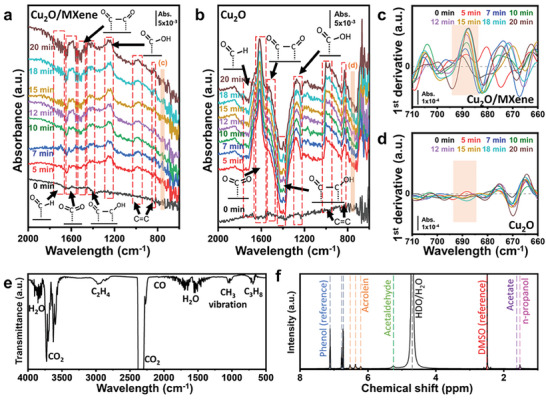
a) and b) ATR‐FTIR spectra for Cu_2_O/MXene and Cu_2_O at varying CO_2_RR time. c) and d) 1st derivative of ATR‐FTIR spectra for Cu_2_O/MXene and Cu_2_O in ranges of 660–710 cm^−1^. e) FT‐IR spectra of gas species from Cu_2_O/MXene CO_2_RR. f) ^1^H NMR spectra of Cu_2_O/MXene. All the tests were conducted at −1.3 V versus RHE.

To determine the mechanism of how the Cu_2_O/MXene affected the selectivity toward C_3_ product in CO_2_RR, *in‐situ* ATR‐FTIR spectroscopy was performed and compared with the Cu_2_O to reveal the coupling effect of the intermediates such as *CO and *OCCO at the interface. As shown in **Figure** **5**a, the peak at 1560–1640 cm^−1^ corresponds to the asymmetric O‐C‐O stretching peak associated with Cu_2_O (Figure 5b) when CO_2_ is adsorbed and the C atom is bound to the metallic atom of the catalyst.^[^ ^34^ ^]^ This O‐C‐O stretching peak remained after 20 min of CO_2_ reduction, indicating that the Cu_2_O anchored on the MXene could continuously and strongly bind the CO_2_ intermediate.^[^ ^35^ ^]^ *CHO peak at 1660–1740 cm^−1^ was expected to produce the C_1_ pathway toward the formation of CH_4_.^[^ ^36^ ^]^ *COOH peak was found at 1210–1280 cm^−1^ which ultimately led to CO.^[^ ^37^ ^]^ Notably, *COOH favors the production of CO, which is not a desirable intermediate for HCOOH formation, as has already been proven by several studies.^[^ ^37^, ^38^ ^]^ C = C peaks were found at 820–850 cm^−1^ and 940−1030 cm^−1^, significant evidence of alkene products including C_2_H_4_.^[^ ^39^ ^]^ Moreover, peaks for CO dimerization were found at 1470−1530 cm^−1^ and 1390–1460 cm^−1^ which belong to *OCCO and *OCCOH onto the Cu_2_O, respectively.^[^ ^36^, ^40^ ^]^ These CO dimerization intermediates triggered the C_2+_ and C_3+_ pathways, as reported in previous studies for the CO_2_RR mechanism to produce C_3_ through the C_1_‐C_2_ coupling step.^[^ ^6^, ^41^ ^]^ The key feature of this mechanism is the high coverage of the C_2_ intermediate, which can be stabilized by a well‐designed catalyst morphology and electronic structure.^[^ ^6d^ ^]^ Here, as the *OCCO and *OCCOH peaks increased with CO_2_ electrolysis time, Cu_2_O (111) strongly bound CO_2_ to C_2_ intermediates and provided catalytic sites for a longer time for additional carbon chain elongation and protonation toward C_3_H_8_, as discussed in the Tafel slopes (Figure 4d; Figure S15, Supporting Information).^[^ ^41^, ^42^ ^]^ To determine the weak C_3_ intermediate peak masked by stronger noise, the first derivative of the ATR‐FTIR was processed, which is a common method used for spectral data processing and interpretation (Figure 5c‐d).^[^ ^43^ ^]^ Compared to Cu_2_O (CuNP‐HT + NH_4_OH), an additional peak at ≈690 cm^−1^ for C‐CH_2_‐C peak was found for Cu_2_O/MXene, confirming that the C_3_ intermediate could generate C_3_H_8_ (propane), n‐C_3_H_7_OH (n‐propanol) or C_2_H_3_CHO (acrolein) during CO_2_ reduction.^[^ ^44^ ^]^ With Cu_2_O, although there are peaks for *CO, *OCCO and *OCCOH, no C‐CH_2_‐C peak at ≈690 cm^−1^ is found, indicating that the C_3_ intermediate is not solely found at the Cu_2_O catalytic site, as well as additional *CO intermediate adsorption site from the MXene is necessary for C_3_ intermediate coupling. Considering that MXene and AT‐MXene could not produce C_2_ products (Figure S13, Supporting Information), the interface between Cu_2_O and MXene in Cu_2_O/MXene was the key site for coupling *C_2_ intermediates and *CO toward C_3_ products, where Cu_2_O provided adsorption sites for *C_2_ intermediates and MXene provided adsorption sites for *CO.

As *CO and *C_2_ bound to couple the carbon chain to C_3+_ products,^[^ ^3^, ^7^, ^45^ ^]^ the gas and liquid products were analyzed by IR and ^1^H NMR to determine the hydrocarbon products (Figure 5e–f). Several peaks representing hydrocarbons from the CO_2_RR were observed. Some unreacted gases, such as CO_2_ and H_2_O, were also found.^[^ ^46^ ^]^ Notably, C‐C‐C skeletal vibration was found at ≈720 cm^−1^, confirming C_3_H_8_ production.^[^ ^47^ ^]^ Through ^1^H NMR, we found additional liquid hydrocarbon products, including C_2_ and C_3_ products such as acetaldehyde, acetate, acrolein, and n‐propanol.^[^ ^48^ ^]^ However, only traceable amounts of liquid products were generated during CO_2_RR.

### 2.3 Proposed Mechanism for Electrochemical CO_2_ Reduction to C_3_H_8_

Based on the above mechanistic analysis, we highlighted the key steps and crucial intermediates in C_3_H_8_ production (Figure S19, Supporting Information). CO_2_ was initially adsorbed onto Cu_2_O/MXene at both Cu_2_O and MXene sites to form *COOH, which underwent further reduction to *CO with H_2_O removal.^[^ ^49^ ^]^ The *CO intermediates on Cu_2_O underwent CO dimerization to *OCCO and were further hydrogenated to form *OCCOH.^[^ ^50^ ^]^ After protonation, *OCCO formed *OCCOH, which is the core step for the formation of C_2_ and C_3_ hydrocarbons, including C_2_H_4_, C_2_H_6_, and C_3_H_7_OH, following sufficient protonation with electron transfer. Furthermore, the stabilized and protonated *C_2_ intermediates on Cu_2_O can be coupled with *CO adsorbed on MXene by NH_4_OH treatment to form the C_3_ intermediate. Additional protonation/electron‐transfer steps, including the hydrogenation of the carbon from the intermediate, can transfer this C_3_ intermediate to the saturated hydrocarbon, i.e., C_3_H_8_.^[^ ^51^ ^]^

The production of C_3_H_8_ on Cu_2_O/MXene benefits from its nanostructure. The catalyst is composed of Cu_2_O with a prevalent Cu_2_O (111) facet, which is in favor of binding *C_2_ intermediates.^[^ ^52^ ^]^ MXene also favors CO_2_ adsorption because the Gibbs free energy of CO_2_ adsorption on MXene is negative, indicating that CO_2_ adsorption on MXene is thermodynamically favorable.^[^ ^53^ ^]^ This hybrid of Cu_2_O and MXene couple *C_2_‐*C_1_ intermediates into C_3_ products at the interface between Cu_2_O and MXene. The NH_4_OH treatment modulated the surface group of MXene to ‐O and ‐OH, which favors the transfer of protons with low H adsorption energy for the protonation of CO_2_RR intermediates.^[^ ^54^ ^]^ Thus, these synergistic effects on the Cu_2_O/MXene structure could increase the C_3_H_8_ production.

**Figure 6:**
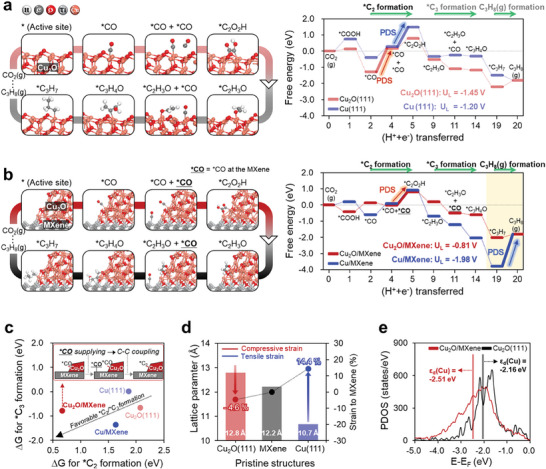
CO_2_RR mechanism and calculated free energy diagram (FED) for C_3_H_8_(g) production in (a) pure Cu_2_O(111) structure and b) 0D/2D heterostructure of Cu_2_O/MXene. c) Correlation between calculated reaction free energies (DG) for *C_2_ and *C_3_ formation through sequential processes of additional *CO supply/C‐C coupling in heterostructures of Cu_2_O/MXene and Cu/MXene and pure Cu_2_O(111) and Cu(111) surfaces. d) Comparison of lattice parameters of pure Cu_2_O(111) and Cu(111) compared to MXene structures with respect to compressive or tensile strain. e) Partial density of state (PDOS) of Cu_2_O/MXene heterostructure and pure Cu_2_O(111) structure for the *d*‐band center (ε_d_) of Cu active sites.

We performed density functional theory (DFT) calculations to gain further insight into the role of MXene on the improved CO_2_RR activity for C_3_H_8_(g) production in the 0D/2D heterostructure composed of Cu_2_O (or Cu)‐anchored MXene compared to pure Cu_2_O and Cu under aqueous condition (**Figure** **6**). Preferentially, we investigated the CO_2_RR activity of pure Cu_2_O(111) and Cu(111) structures through their limiting potential (U_L_) evaluated by the free energy diagram (FED) constructed for experimentally observed intermediates in the CO_2_RR mechanism depicted in Figure 6a and Figure S19 (Supporting Information) (Details of calculated thermodynamic values for FED are listed in Tables S6 and S7, Supporting Information).^[^ ^55^ ^]^ The calculated FED in Figure 6a shows that the potential determining steps (PDS) correspond to C_1_‐C_1_ coupling (*CO+*CO → *C_2_O_2_H) and additional *CO supply (*CO → *CO+*CO) steps with U_L_ values of −1.45 V and −1.20 V at Cu(111) and Cu_2_O(111), respectively. The difference in PDS steps for pure Cu_2_O(111) and Cu(111) structures can be interpreted by the binding energy of the intermediate. Cu(111) surface has a higher energy barrier in C_1_‐C_1_ coupling for *C_2_O_2_H formation due to the relatively weak binding energy of intermediates, especially *C_2_O_2_H and *CO. In contrast, Cu_2_O(111) provides a very strong *CO binding at a well‐known coordinatively unsaturated Cu site (Cu_cus_), and additional *CO supply for *C_2_O_2_H formation cannot be easily achieved because neighboring *CO binding sites are coordinatively saturated Cu site (Cu_css_) to which *CO cannot stably bind, and instead Cu_cus_ sites are located far apart, as shown in Figure S21 (Supporting Information).^[^ ^56^ ^]^ Our results imply that *C_2_O_2_H formation is a thermodynamically unfavorable process in pure Cu_2_O(111) and Cu(111) structures, resulting in low *C_3_ intermediate selectivity. Therefore, MXene can be expected to play a positive role in this *C_2_O_2_H formation process.

To verify the role of MXene considering the experimentally characterized 0D/2D heterostructures (Figure 1) of Cu_2_O/MXene, we first constructed thermodynamically stable 2D/2D heterostructures of Cu_2_O and MXene, and then manipulated the Cu_2_O interface to expose the MXene surface to become a 0D/2D heterostructure, as shown in Figure 6b.^[^ ^57^ ^]^ Note that 2D/2D and 0D/2D heterostructure construction processes and their formation energies are described in Figure S21 and Table S8 (Supporting Information). Using the well‐established 0D/2D heterostructures, we constructed FED to clarify the effect of MXene incorporation on the PDS and U_L_ values (Figure 6b). In the case of Cu_2_O/MXene heterostructure, the FED shows improved *C_2_ intermediate selectivity compared to Cu_2_O(111) by lowering U_L_ value from −1.45 V to −0.81 V. It is also seen that the overall binding energy of intermediates on Cu_2_O/MXene is weakened by MXene incorporation, ultimately overcoming the strong *CO binding problem that caused the high energy barrier on the pure Cu_2_O(111) surface.^[^ ^58^ ^]^ In addition, MXene can act as an additional *CO supplier for *C_2_O_2_H formation through a thermodynamically spontaneous process due to MXene's preference for *CO, as shown in Figure S23 and Table S9 (Supporting Information). In contrast, for the FED of Cu/MXene heterostructure, the higher energy barrier for *C_2_O_2_H formation at the C_1_‐C_1_ coupling step can be reduced by the overall enhanced binding energy of the intermediate. Subsequently, both Cu_2_O/MXene and Cu/MXene heterostructures facilitate the formation of *C_2_O_2_H and enable further reduction processes as a spontaneous downhill reaction to produce C_3_ compounds such as C_3_H_8_ through *C_2_‐*C_1_ coupling. Moreover, the correlation analysis between calculated reaction free energies for *C_2_O_2_H and *C_3_H_4_O formation through sequential processes of additional *CO supply and C‐C coupling in Figure 6c and Table S10 (Supporting Information) clearly shows that the MXene incorporation plays an important role in promoting the formation reactions of *C_2_O_2_H and *C_3_H_4_O.^[^ ^59^ ^]^ Therefore, Cu_2_O/MXene can proceed CO_2_RR to C_3_H_8_(g) production without specifically high energy barrier. However, Cu/MXene cannot produce the final C_3_H_8_(g) compound due to the enhanced intermediate binding energy. Especially, the strong binding energy of Cu/MXene for *C_3_H_7_ results in a very high energy barrier for C_3_H_8_(g) formation, thus increasing the U_L_ value from −1.20 V to −1.98 V compared to pure Cu(111).

The conflicting behavior of intermediate binding energy can be comprehended by lattice parameter mismatch in the Cu_2_O/MXene and Cu/MXene heterostructures, as analyzed in Figure 6d. Cu_2_O and Cu in the heterostructure with MXene undergo compressive (−4.6%) and tensile (+14.4%) strain, respectively. Cu in the Cu/MXene heterostructure suffers severe lattice strain, and the extremely large tensile strain on Cu induces strong binding energy of intermediate to stabilize the strained structure through intermediate binding. For Cu_2_O in the Cu_2_O/MXene heterostructure, the weakened binding energy of the intermediate can be explained by electronic structure modulation, i.e., *d*‐band center shift. The binding strength of adsorbents is closely related to the filling of the anti‐bonding state near the Fermi level, which is estimated by the *d*‐band center (ε_d_) theory of Hammer and Norskov.^[^ ^60^ ^]^ The increased filling of the anti‐bonding state leads to weak binding energy of adsorbents. The partial density of states (PDOS) analysis of the *d*‐orbital of Cu (ε_d_(Cu)) in Figure 6e reveals that ε_d_(Cu) of each Cu_2_O(111) and Cu_2_O/MXene are located at −2.16 eV and −2.51 eV, respectively. Here, the downshifted ε_d_(Cu) of Cu_2_O/MXene position compared to ε_d_(Cu) of Cu_2_O(111) proves that the binding energy of intermediates in Cu_2_O/MXene is relatively weak. Consequently, our theoretical calculations clearly show that the incorporation of MXene, especially into the Cu_2_O/MXene heterostructure plays a crucial role in the improvement of CO_2_RR catalytic activity up to C_3_H_8_(g) production by regulating the electronic structure and promoting the sequential processes of *CO supplying/C‐C coupling.

The intermediates involved in this mechanism are more complicated than those suggested in our study as 3 CO_2_ molecules with 20 electrons are required for C_3_H_8_ production. Nevertheless, the mechanism described in our study offers opportunities for the design of advanced catalysts for the efficient production of C_3_H_8_, with a pathway for electrochemical CO_2_ reduction and protonation of the hydrocarbon intermediates.

## 3 Conclusion

In summary, we designed Cu_2_O/Ti_3_C_2_T_x_ MXene, hybridized oxide‐derived Cu (Cu_2_O) on OH‐modulated MXene by NH_4_OH treatment and achieved highly active and selective production of C_3_H_8_ via electrochemical CO_2_ reduction reaction in aqueous electrolyte. ATR‐FTIR showed that Cu_2_O can strongly bind and stably preserve the reaction intermediates on the surface, but additional active sites are required for *C_2_‐*C_1_ coupling toward C_3_ hydrocarbons. Thus, owing to the synergistic effects of Cu_2_O and MXene, the interface between Cu_2_O and MXene is a key factor for the CO_2_ reduction to C_3_H_8_, where Cu_2_O binds *C_2_ intermediates and MXene binds *CO for C_3_ coupling with further efficient protonation for C_3_H_8_ production. In detailed characterizations, Cu_2_O/MXene exhibited a remarkable FE enhancement toward C_3_H_8_ (≈3.3%), representing a 26‐fold increase compared to Cu/MXene without the aid of carbon sources neither on the surface on the catalyst nor the ionomer. DFT calculations also highlight the effective pathway for electrochemical CO_2_ reduction and protonation of hydrocarbon intermediates to produce C_3_H_8_ in the presence of the interface between Cu_2_O and MXene. This study not only provides evidence of the reaction process in the selective electrochemical reduction of CO_2_ to C_3_H_8_ but also suggests a rational electrocatalyst design strategy for CO_2_RR. This strategy involves multi‐carbon coupling as well as protonation to produce high‐energy‐density products, including hydrogen‐rich hydrocarbons.

## 4 Experimental Section

### Synthesis of Ti_3_C_2_T_x_ MXene

2D Ti_3_C_2_T_x_ MXene nanosheets were prepared by a typical etching method previously reported with some modifications.^[^ ^14^ ^]^ Initially, 2.0 g of the MAX powder (Ti_3_AlC_2_) was added to 20 mL of an aqueous HF solution in a Teflon liner and stirred for 36 h at 50 °C to selectively etch the Al layer from the MAX powder. The obtained suspension was washed several times with deionized water (DI water) via centrifugation to remove the acidic solvent. The precipitated samples were then collected and freeze‐dried. After freeze‐drying, the 1.0 g of MXene powder was mixed with 12 mL of DMSO and stirred for 18 h at room temperature for intercalation. After intercalation, a tip sonicator was used in an ice‐water bath for 4 h for delamination. The resulting solution was centrifuged several times with DI water to remove the remained DMSO. Finally, the obtained MXene was dispersed in DI water and stored in the fridge until use.

### Synthesis of Cu Nanoparticles

First, 30 mmol of 1‐octadecene, 4 mmol of oleic acid, and 8 mmol of 1,5‐pentanediol were mixed and heated at 130 °C for 30 min under an N_2_ atmosphere in a two‐necked round bottom flask to form solution A. Another bottle of 1 mmol Cu(acac)_2_ into 5 mmol oleylamine was heated at 70 °C for 30 min with stirring to form solution B. Subsequently, solution B was injected into solution A and heated at 200 °C for 2 h under stirring. After heating at 200 °C, the obtained solution was then rapidly cooled. To remove organic impurities, the sample was washed and centrifuged several times with hexane and IPA, then freeze‐dried. Finally, the products obtained as a powder were denoted as CuNPs.

### Synthesis of Cu_2_O/MXene

The as‐prepared MXene solution was diluted to 1 mg mL^−1^ in DI water and sonicated for 30 min to prevent restacking. The synthesis of Cu_2_O/MXene adopted a hydrothermal method. 6 mg of Cu NPs and 4 mmol of ammonia solution (NH_4_OH) were added into 25 mL of the MXene solution and kept at 70 °C for 30 min under N_2_ to inhibit further oxidation of MXene. For comparison, different mass ratios of CuNPs to MXene were prepared: 2:25, 6:25, and 10:25 wt./wt. (denoted as Cu_2_O/MXene 2:25, 6:25, and 10:25, respectively). The mixture was then ultrasonicated at room temperature for 1 h. After sonication, the obtained Cu_2_O/MXene sample was washed several times with DI water, centrifuged, and vacuum dried to prevent further oxidation. Cu/MXene was prepared following the same procedure as for Cu_2_O/MXene, except for adding 4 mmol of NH_4_OH. Additionally, MXene subjected to a hydrothermal process with NH_4_OH treatment (AT‐MXene) was prepared following the same procedure as for Cu_2_O/MXene, except for adding CuNPs.

### Electrochemical Measurements

The electrochemical CO_2_ reduction reaction (CO_2_RR) measurements were conducted by a three‐electrode configuration using an electrochemical working station (Gamry Reference 600+). To prepare the working electrode, 2 mg of catalyst was dispersed in 0.5 mL isopropyl alcohol (IPA) with a sonicator. After the sonication, 5 µL of Nafion (5 wt.%) was additionally mixed with the catalyst solution and further ultrasonicated for 30 min to obtain a catalyst ink. The 40 µl of ink was then dropped onto the carbon cloth with a geometric area of 1 cm^2^ and dried in the vacuum oven. The carbon cloth was pretreated by soaking it in 3 M HCl for 15 min and then washed several times with DI water and ethanol, followed by N_2_ blowing until dry. The Pt plate and Ag/AgCl (3 M NaCl) electrode served as the counter and reference electrodes, respectively. As an electrolyte, CO_2_ saturated with 0.1 M KHCO_3_ (pH = 6.8) was prepared and kept at a CO_2_ flow rate of 20 sccm using a mass flow controller during electrochemical CO_2_RR measurements. LSV was performed at a scan rate of 5 mV s^−1^. All potentials were reported with respect to the RHE with the *iR* correction. Electrochemical impedance spectroscopy (EIS) was conducted in the frequency range of 10^5^–10^−1^ Hz and amplitude of 5 mV at −1.0 V versus RHE. The electrochemical double‐layer capacitance method was used for the electrochemical active surface area (ECSA) measurements, which were extracted from the cyclic voltammograms (CV) at different scan rates (2, 5, 10, 20, and 25 mV s^−1^) in the non‐Faradaic region. Note that all electrochemical data (except stability testing) were repeated more than three times, with error bars representing the standard deviation of the data.

### Computational Details

All density functional theory (DFT) calculations were performed with the Vienna Ab initio Simulation Package (VASP 5.4.4).^[^ ^61^ ^]^ The Projector Augmented Wave (PAW) method^[^ ^61^, ^62^ ^]^ was employed and exchange‐correlation interactions were treated through Perdew‐Burke‐Ernzerohf (PBE)^[^ ^63^ ^]^ functional under the generalized gradient approximation (GGA). Monkhorst‐Pack k‐point meshes of 2 × 4 × 1 and 1 × 4 × 1 were used in each primitive lattice vector of the reciprocal space for geometry optimization of pure Cu_2_O(111) and Cu(111) and Cu_2_O (or Cu)/MXene heterostructures, respectively with Brillouin zone sampling.^[^ ^64^ ^]^ And DFT‐D3 dispersion correction method was used to reflect the non‐bonding interactions correlation in the Cu_2_O/MXene system.^[^ ^63^, ^65^ ^]^ Lattice constants and internal atomic positions were fully optimized using a plane‐wave cutoff energy of 500 eV and spin‐polarized calculations until the residual forces were less than 0.04 eV Å^−1^. Additional description of the structural information and CO_2_RR catalytic activity evaluation is described in the Supporting Information.

## Conflict of Interest

The authors declare no conflict of interest.

## Author Contributions

J.Y.K. and W.T.H. contributed equally to this work. J.Y.K. designed the experiment and methodology and conducted the formal analysis and investigations. W.T.H. performed the data curation, material synthesis, characterization, and investigation. T.K.C.P. performed the methodology, assisted with investigations, and contributed to the initiation of this work. S.C.C. conducted the DFT calculation. B.K. and U.B. assisted with the investigation and methodology. H.‐S.O., J.H.K., X.Y., C.‐H.C., and J.P. conducted the validations, assisted with the formal analysis, and conducted funding acquisition. S.U.L. supervised the DFT calculation, provided the resources and revised the manuscript. C.‐H.C. provided the resources, conducted the investigation and funding acquisition, and assisted with the formal analysis. J.K.K. conceptualized this work, supervised this project, performed project administration, and conducted funding acquisition. All authors contributed to the writing, revising, and editing of this manuscript.

## Supporting information

Supporting Information

## Data Availability

The data that support the findings of this study are available from the corresponding author upon reasonable request.
