# Supplementary material for: Proton‐Coupled Electron Transfer on Cu2O/Ti3C2Tx MXene for Propane (C3H8) Synthesis from Electrochemical CO2 Reduction
Source: Adv Sci (Weinh). 2024 Aug 19;11(39):2405154. doi: 10.1002/advs.202405154 (PMC11497005; doi:10.1002/advs.202405154)
Supplement: Supplementary file 1 — Supporting Information [file ADVS-11-2405154-s001.docx]

**Supplementary Information**

**Proton-coupled electron transfer on Cu_2_O/Ti_3_C_2_T_x_ MXene for propane (C_3_H_8_) synthesis from electrochemical CO_2_ reduction**

Jun Young Kim, Won Tae Hong, Thi Kim Cuong Phu, Seong Chan Cho, Byeongkyu Kim, Unbeom Baeck, Hyung-Suk Oh, Jai Hyun Koh, Xu Yu, Chang Hyuck Choi, Jongwook Park*, Sang Uck Lee,* Chan-Hwa Chung, and Jung Kyu Kim*

J. Y. Kim, W. T. Hong, T. K. C. Phu, S. C. Cho, B. Kim, U. Baeck, S. U. Lee, C.-H. Chung, J. K. Kim

School of Chemical Engineering, Sungkyunkwan University (SKKU), 2066, Seobu-ro, Jangan-gu, Suwon 16419, Republic of Korea.

E-mails: legkim@skku.edu (J. K. Kim), suleechem@skku.edu (S. U. Lee)

J. K. Kim

SKKU Advanced Institute of Nano Technology (SAINT), Sungkyunkwan University, 2066 Seobu-ro, Suwon 16419, Republic of Korea.

J. H. Koh, H.-S. Oh

Clean Energy Research Center, Korea Institute of Science and Technology (KIST), Hwarang-ro 14-gil 5, Seongbuk-gu, Seoul 02792, Republic of Korea.

X. Yu

School of Chemistry and Chemical Engineering, Yangzhou University, Yangzhou, 225002 P. R. China.

C. H. Choi

Department of Chemistry, Pohang University of Science and Technology (POSTECH), Pohang, 37673, Republic of Korea.

Institute of Convergence Research and Education in Advanced Technology (I-CREATE), Yonsei University, Seoul, 03722, Republic of Korea.

J. Park

Integrated Engineering, Department of Chemical Engineering, Kyung Hee University, Gyeonggi, 17104, South Korea.

E-mail: jongpark@khu.ac.kr (J. Park)

**Experimental Details**

**Chemicals**

Hydrofluoric acid (HF, 48 wt%), copper (II) acetylacetonate (Cu(acac)_2_, ≥ 99.9%), oleylamine (≥ 98%), oleic acid (≥ 90%), 1-octadecene (≥ 99.0%), 1,5-pentanediol (96%), ammonia solution (NH_4_OH, 25%), potassium bicarbonate (KHCO_3_, powder, 99.7%), D_2_O (99.9% deuterium atom), and potassium chloride (KCl, powder, 99.0−100.05%) were purchased from Sigma-Aldrich and used as received. Dimethyl sulfoxide (DMSO, anhydrous, ≥99.9%) was purchased from Thermo Scientific. Ti_3_AlC_2_ (MAX) powder was purchased from SY Innovation. Nafion solution (5 wt.%) was purchased from Alfa Aesar. Isopropyl alcohol (IPA, 99.5%) was purchased from Junsei Chemical. Hydrochloric acid (HCl, 37%) and ethanol (99.5%) were purchased from Samchun Chemical.

**Products Analysis of Electrochemical CO_2_ Reduction**

CO_2_ reduction tests were conducted at different cell potentials (−0.9, −1.1, −1.3, and −1.5 V vs. RHE) for 1 h. The resultant gaseous product was collected in a Tedlar bag and the liquid product was obtained from the catholyte.

0.5 mL of the gas product was taken and injected into a gas chromatograph (GC, YL 6500GC) equipped with a thermal conductivity detector (TCD) and methanizer-connected flame ionization detector (methanizer/FID).

The Faradaic efficiency for a specific product is calculated with the following equation:

$$FE(\%)=\frac{z\times n\times F}{Q}\times100$$

where *z* is the number of electrons exchanged, *n* is the number of moles of a specific product, *F* is the Faraday constant (F = 96485 C/mol), and *Q* is the charge passed. The partial current density for a specific product can be calculated by multiplying the total current density by the Faradaic efficiency of the specific product.

For liquid product analysis, the catholyte after the CO_2_RR was analyzed using ^1^H nuclear magnetic resonance (NMR 700 MHz, ADVANCEIII700). 400 μL of the catholyte was mixed with an internal standard consisting of 35 μL (10 mM) DMSO and 50 mM phenol in 100 μL D_2_O. The Faradaic efficiency of the liquid product was calculated with the following equation below:

$$FE_{liquid}\left( \% \right)=(c_{liq}\times V\times z\times F)/Q\times100$$

where *c_liq_* is the concentration of the liquid, *V* is the volume of the electrolyte, and *Q* is the quantity of electric charge integrated by the potentiostat.

The turnover frequency (TOF) for a given product was calculated as:

$$TOF=\frac{I_{product}/zF}{m_{cat}\times\omega/M}\times3600$$

where *I_produc_*_t_ (A) is the partial current for the product, *m_cat_* (g) is the catalyst mass on the carbon cloth, *ω* is the loading in the catalyst and *M* is the atomic mass of the catalyst that participated in the electrolysis. The TOFs were determined by normalizing the current density to the electrochemical surface area, listed in Table S5. Note that all electrochemical data (except stability testing) were repeated more than three times, with error bars representing the standard deviation of the data.

**Catalyst Characterizations**

The morphologies and structures of the catalysts were investigated using a Cs-corrected scanning transmission electron microscope (Cs-STEM, JEM-ARM200F) at an accelerating voltage of 200 kV equipped with an energy dispersive X-ray spectrometer and an electron energy-loss spectrometer. X-ray diffraction (XRD) patterns were measured on an X’Pert PRO multipurpose x-ray diffractometer equipped with a Cu Kα source. The diffraction patterns were collected from 5° to 90° at a scan rate of 2°/min. X-ray photoelectron spectroscopy (XPS) was performed on ESCALAB 250Xi (Thermo Fisher Scientific) with monochromatic Al-Kα radiation operated at 150 W. AES was tested using a PHI 700 (ULVAC-PHI). Raman spectroscopy (DXR2xi, Thermo Fisher Scientific) was employed to collect spectra with a 532 nm laser as the excitation source from 150 to 2000 cm^−1^. The FT-IR spectra were collected at the resolution of 4 cm^−1^ on a Bruker IFS-66/S, TENSOR27 in the 400-4000 cm^−1^ region. Attenuated total reflection-Fourier transformed infrared (ATR-FTIR) spectra were recorded *in-situ* using a Fourier-transform infrared (FT-IR) spectrometer (JASCO, FT/IR-4700) equipped with a mercury-cadmium-telluride (MCT) detector. The *in-situ* cell was composed of ZnSe crystal for the IR analysis, and CO_2_ gas flowed during the reaction. The light was irradiated and the FT-IR spectra were recorded as a function of time to investigate the dynamics of the electrocatalytic conversion reaction. Cu K-edge absorption spectroscopy (XAS) was performed using hard X-rays at the 1D beamline of the (R-XAS) at the Korea Institute of Science and Technology (KIST). X-ray adsorption near-edge structure (XANES) and extended X-ray absorption fine structure (EXAFS) were employed to analyze the standard samples, and the catalyst was also analyzed through the measured spectra.

**CO_2_RR catalytic activity evaluation**

For a reasonable density functional theory (DFT) simulation to describe the CO_2_RR catalytic activity, we employed the computational hydrogen electrode (CHE) model proposed by Noskov et al.,^[1]^ which treats the pair energy consisting of a proton (H^+^) and an electron as calculated by the half of the H_2_(g) molecule based on the following equation (applied potential (U) = 0 V, pH =0, p = 1 atm, T = 298K).

| ${G(H}^{+}+e^{-})= G\left( 1/2H_{2} \right), \Delta G^{0}=0 eV$ |  |
| --- | --- |

The CHE model with its equilibrium potential is related to the thermodynamics of the reaction. Notably, when the adsorbate is adsorbed to the surface, adsorption Gibbs free energy ($\Delta G_{\mathrm{ads}^{*}}$) is defined as

| $\Delta G_{\mathrm{ads}^{*}}= \Delta E_{\mathrm{ads}^{*}}+\Delta ZPE-T\Delta S+\Delta G_{U}+\Delta G_{\mathrm{pH}}$ |  |
| --- | --- |

where $\Delta E_{\mathrm{ads}^{*}}$ is the adsorption energy, $\Delta ZPE$ is the change of zero-point energy after reaction intermediates adsorption to the surface, $T\Delta S$ is also the change of entropy with temperature (T = 298.15K),^[2]^ $\Delta G_{U}$ is correction term related to applied bias, $\Delta G_{\mathrm{pH}}$ is an experimental term to consider proton concentration, which described as 2.303 $\times$ k_B_T $\times$ pH (=0.059 $\times$ pH), and k_B_ is the Boltzmann constant.^[3]^ The adsorption energy ($\Delta E_{\mathrm{ads}^{*}}$) is also expressed as

| $\Delta E_{\mathrm{ads}^{*}}=E_{\mathrm{ads}^{*}}-E_{*}-E_{\mathrm{ads}}$ |  |
| --- | --- |

where $E_{\mathrm{ads}^{*}}$ is the total energy of the adsorbate adsorbed on the active site (*) of the target system, $E_{*}$ is the total energy of the surface structure, and $E_{\mathrm{ads}^{*}}$ is the energy of adsorbate. In addition, the limiting potential (U_L_) is an important descriptor for determining the catalytic activity because the U_L_ value means the required potential to occur a thermodynamically spontaneous reaction to overcome the bottleneck of the multi-step electrochemical reaction. The U_L_ is expressed following the equation by

| $U_{L}=-\Delta G_{\max}/e$ |
| --- |

where the $\Delta G_{\max}$ is the maximum free energy change during the overall catalytic process between the following reaction steps, $\Delta G_{\mathrm{COO}H^{*}}$, $\Delta G_{\mathrm{CO}^{*}}$, $\Delta G_{\mathrm{CO}^{*}+CO^{*}}$, $\Delta G_{C_{2}O_{2}H^{*}}$, $\Delta G_{C_{2}H_{3}O^{*}}$, $\Delta G_{C_{2}H_{3}O^{*}+CO^{*}}$, $\Delta G_{C_{3}H_{4}O^{*}}$, $\Delta G_{C_{3}H_{7}^{*}}$, and $\Delta G_{C_{3}H_{8}(g)}$.

**Description of structural properties**

**Surface modeling and evaluating surface energy of pure Cu_2_O(111) and Cu(111) surface structures**

The pure Cu_2_O(111) and Cu(111) surface structures have vacuum space in the z-direction set to 15 Å to avoid interactions between layers and the bottom two layers of them were fixed to represent its bulk properties (Figure S22). Then, to evaluate the stability of surfaces, we calculated surface energy ($\sigma_{\mathrm{surface}}$) which is defined by

| $\sigma_{\mathrm{surface}}=(E_{\mathrm{surface}}-E_{\mathrm{bulk}}\times N)/(2A)$ |
| --- |

where $E_{\mathrm{surface}}$ is the total energy of the fully relaxed surface structures (Cu_2_O(111) and Cu(111)), $E_{\mathrm{bulk}}$ is the total energy of Cu_2_O or Cu bulk energy, $N$ is the corresponding number of bulk units in the surface structure, and A is the surface area.

**Designing process of 2D/2D and 0D/2D heterostructures of Cu_2_O (or Cu)/MXene**

To compare CO_2_RR activity depending on the establishment of the heterostructure, we designed a 2D/2D Cu_2_O (or Cu)/MXene heterostructure with minimizing lattice mismatch between Cu_2_O (or Cu) and MXene. To examine the interfacial configurations along the x and y axes at certain z-axis values (Figure S21), we conducted systematic screening for the Cu_2_O (or Cu)/MXene heterostructure. Among the proper candidates for heterostructures, we evaluated their thermodynamic stability by heterostructure formation energy ($E^{f}$), which is defined as

| $E^{f}={(E}_{Cu_{2}O(Cu)/MXene}-(E_{\mathrm{Cu}_{2}O(Cu)}+E_{\mathrm{MXene}}))/A$ |
| --- |

where $E_{Cu_{2}O(Cu)/MXene}$ is the total energy of fully relaxed Cu_2_O (or Cu)/MXene heterostructure, $E_{\mathrm{Cu}_{2}O(Cu)}$ and $E_{\mathrm{MXene}}$ are the energy of Cu_2_O (or Cu) and MXene structure, respectively, $A$ is the area of heterostructure.


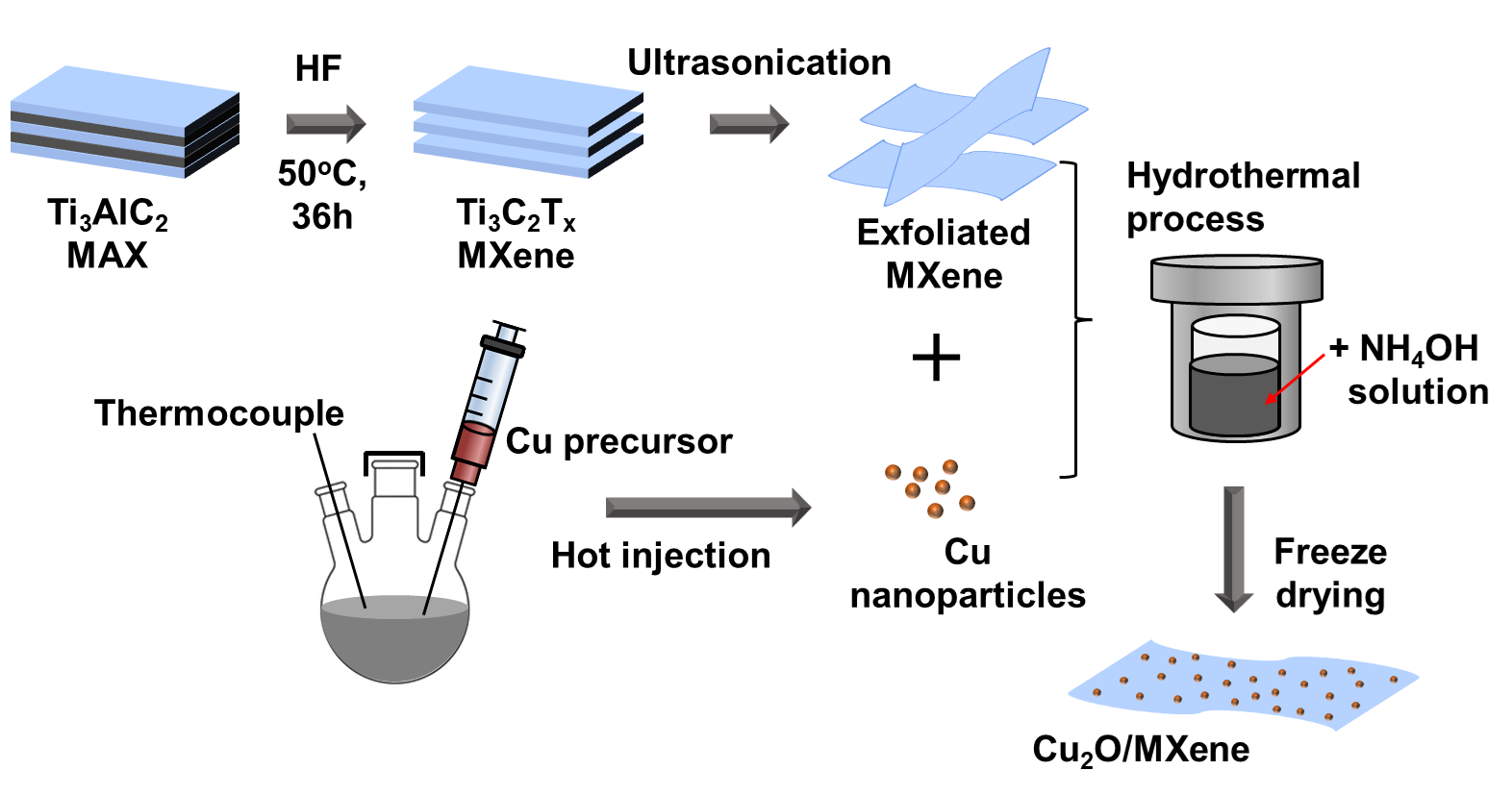


**Scheme S1.** Schematic illustration of Cu_2_O/MXene synthesis.

**
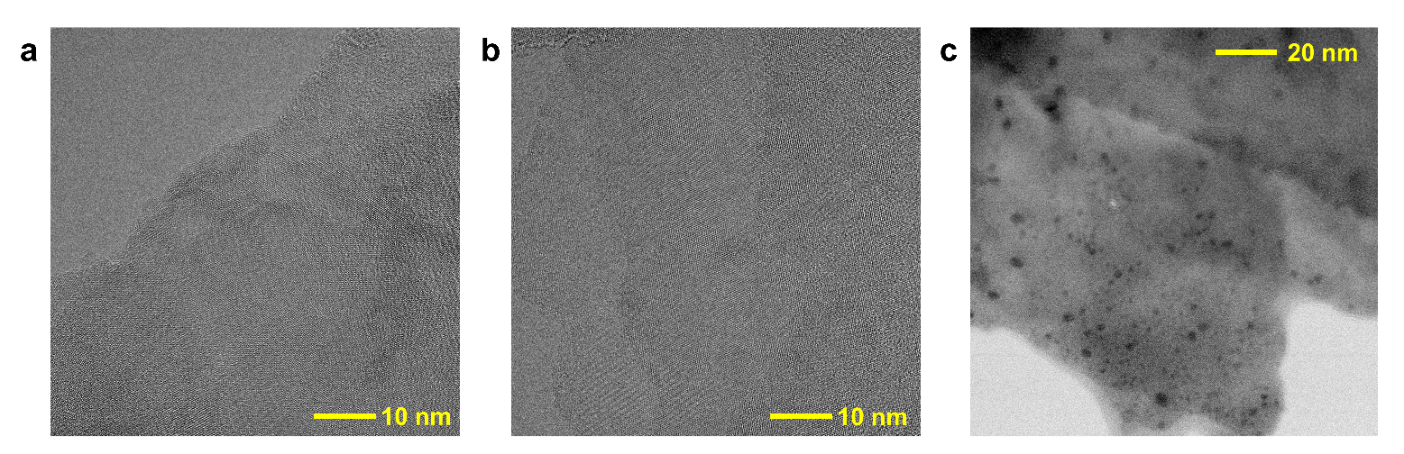
**

**Figure S1.** TEM images of (a) MXene, (b) NH_4_OH-treated MXene (AT-MXene), and (c) HAADF image Cu_2_O/MXene.


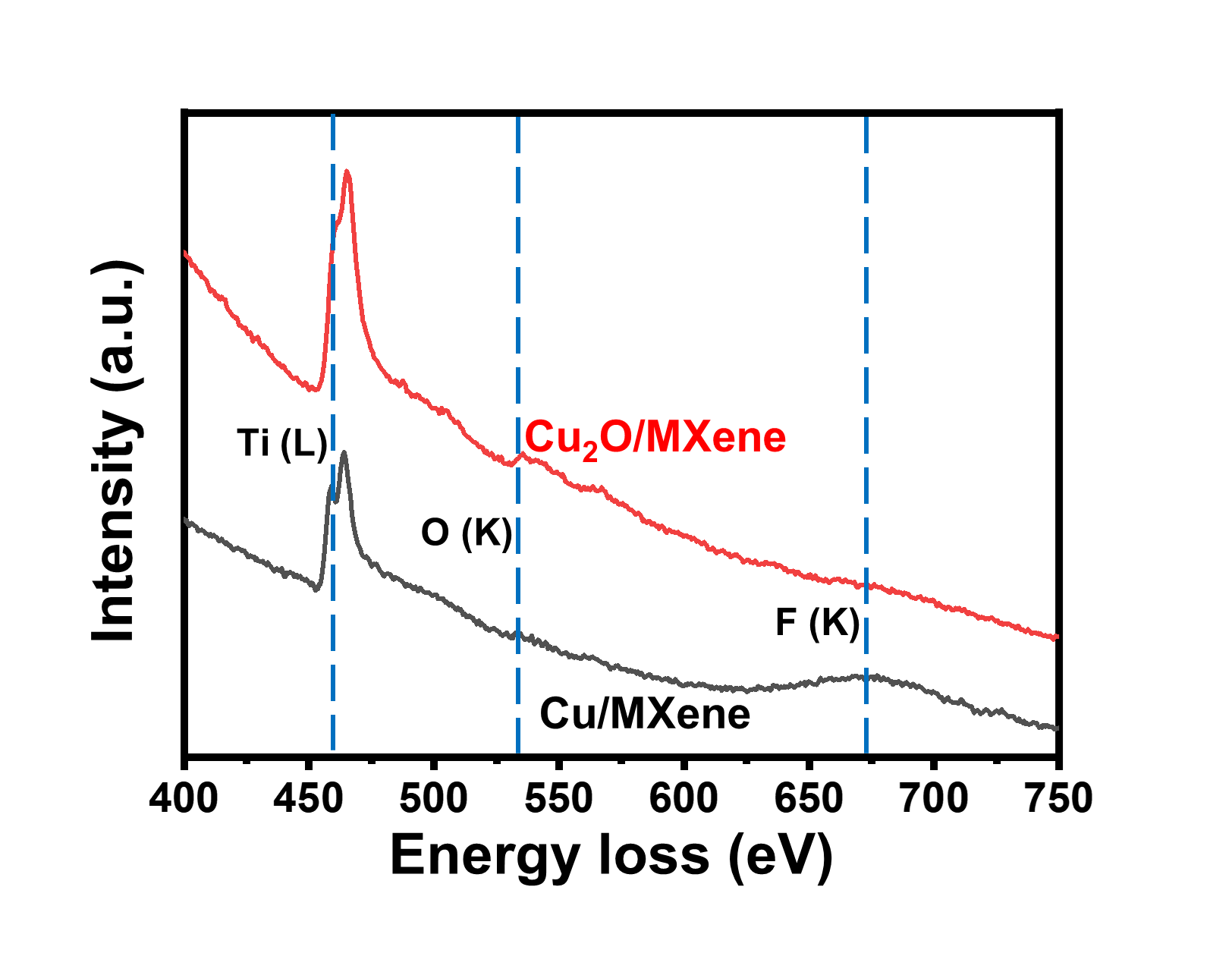


**Figure S2.** EELS spectrum of Cu_2_O/MXene and Cu/MXene.


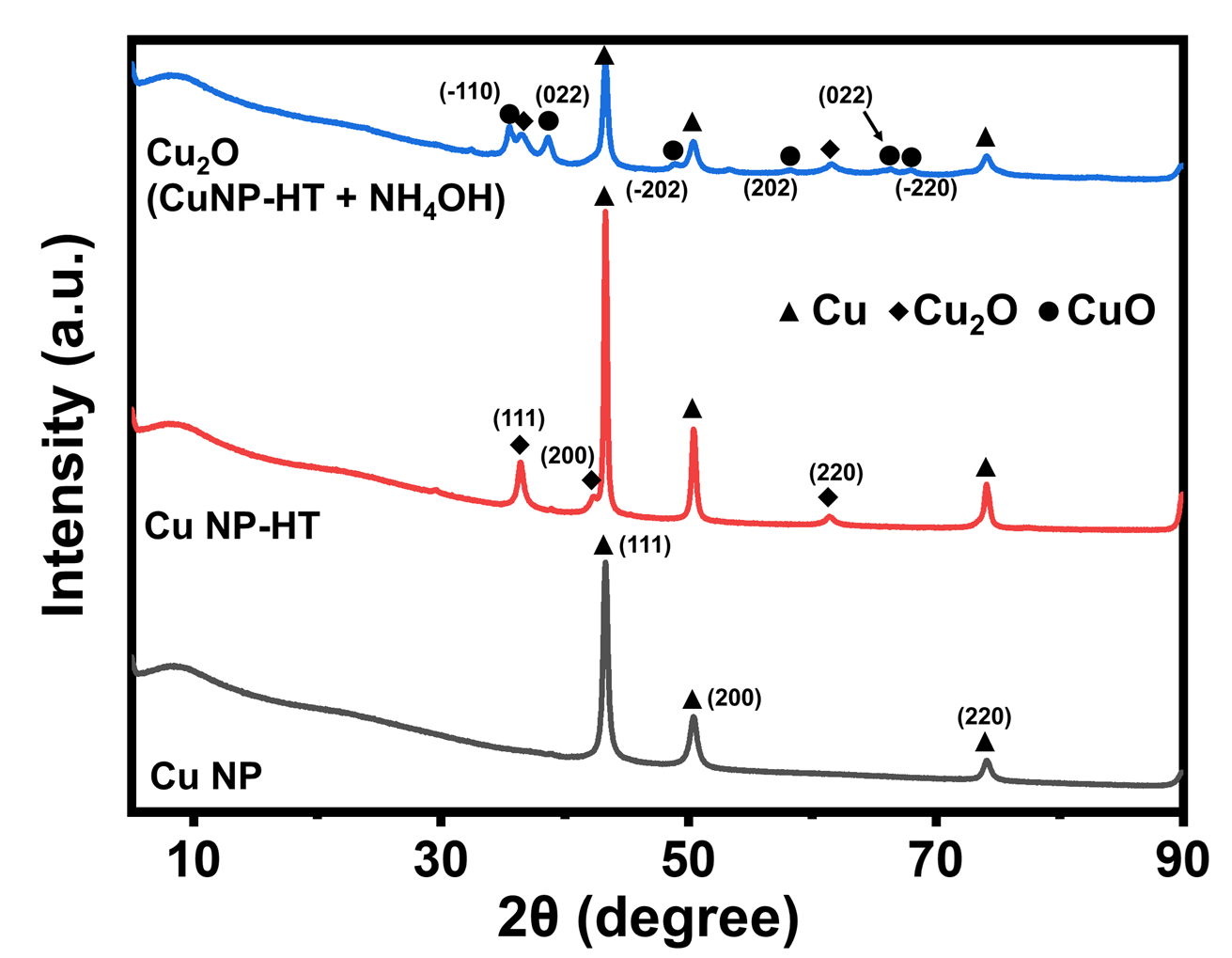


**Figure S3.** XRD of Cu nanoparticles with three different treatments after hot injection.

In the absence of MXene, CuNPs are further oxidized to form CuO when they are treated by same manner with Cu_2_O/MXene.


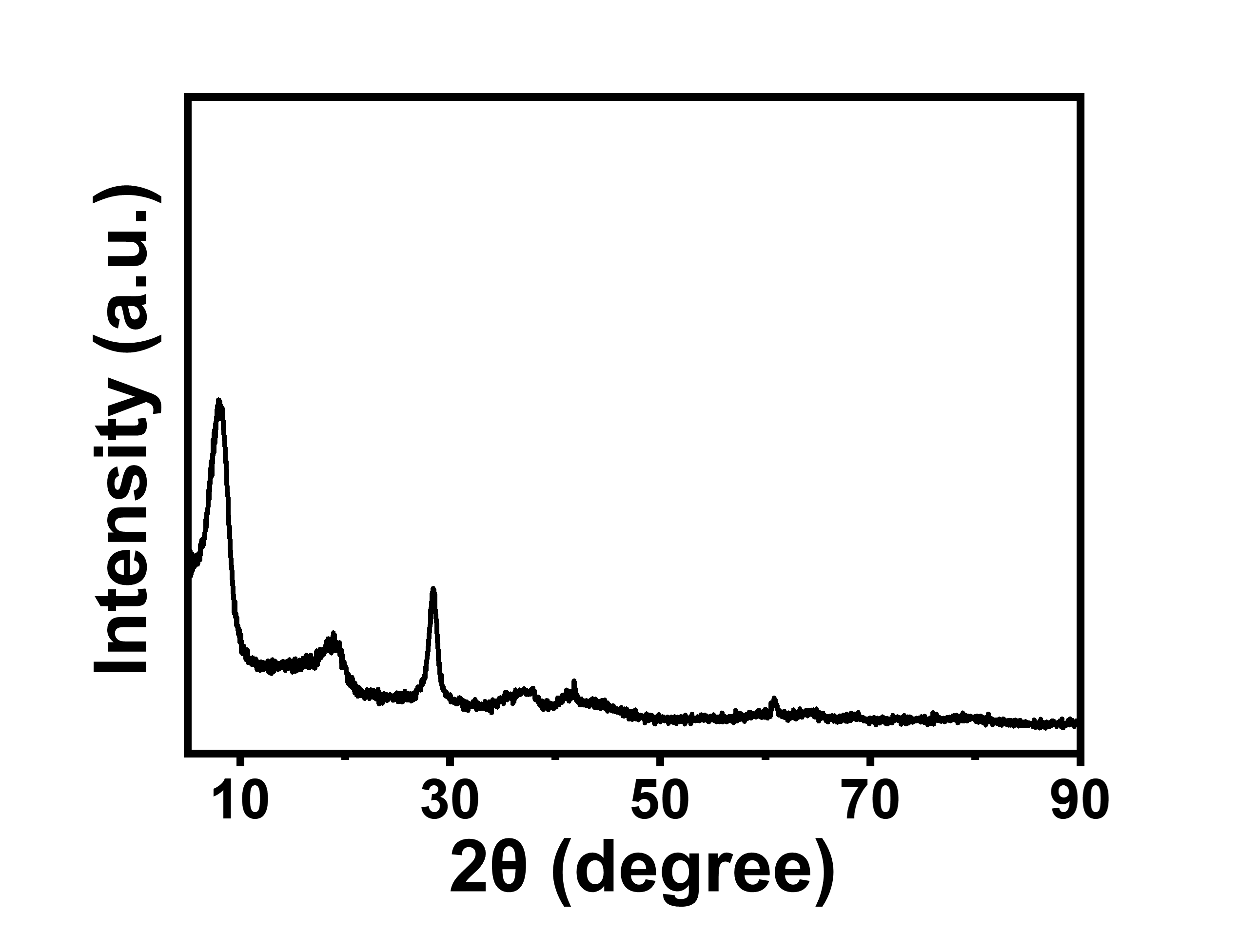


**Figure S4.** XRD of AT-MXene.


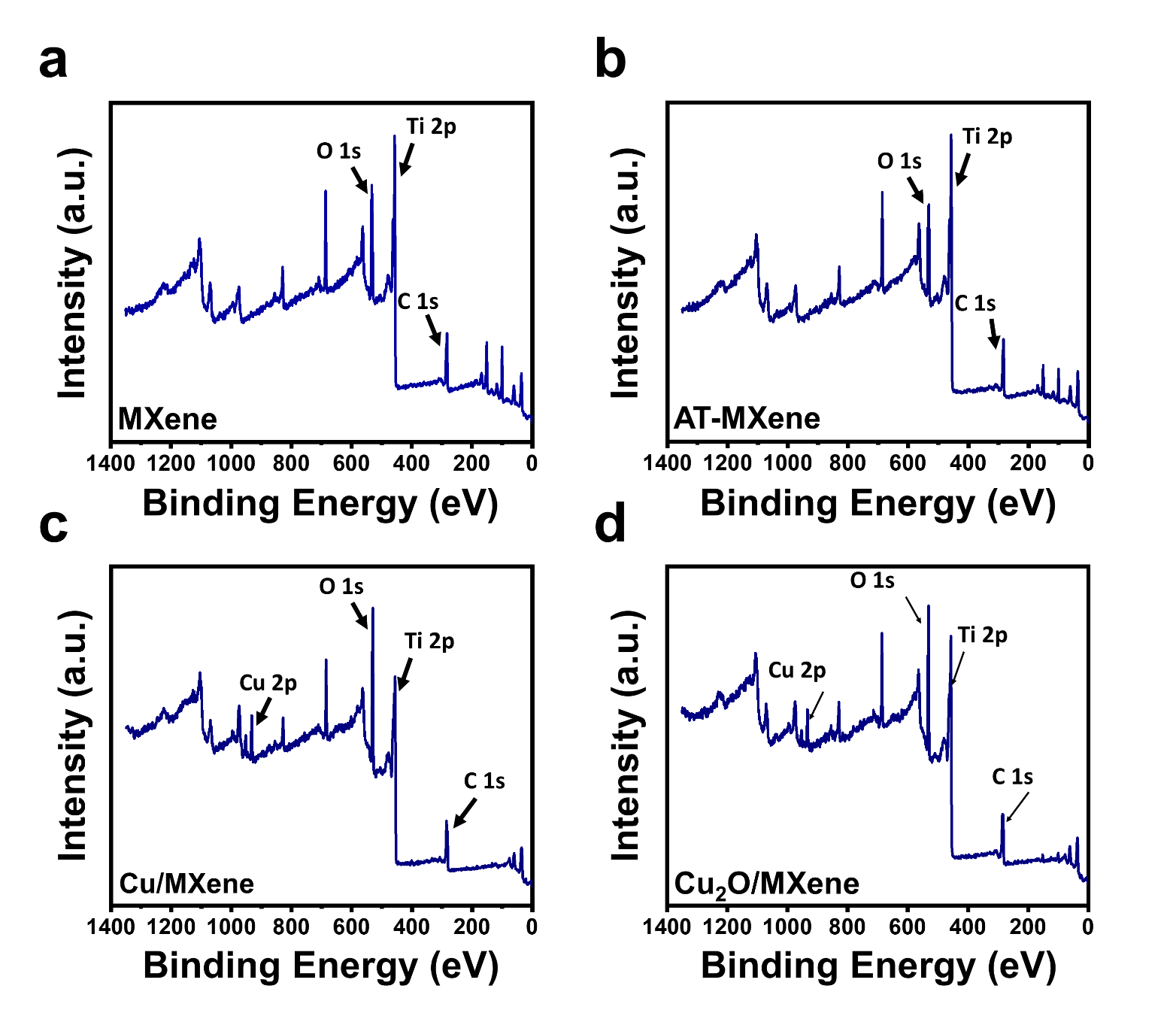


**Figure S5.** Survey XPS spectra of (a) MXene, (b) AT-MXene, (c) Cu/MXene, and (d) Cu_2_O/MXene.


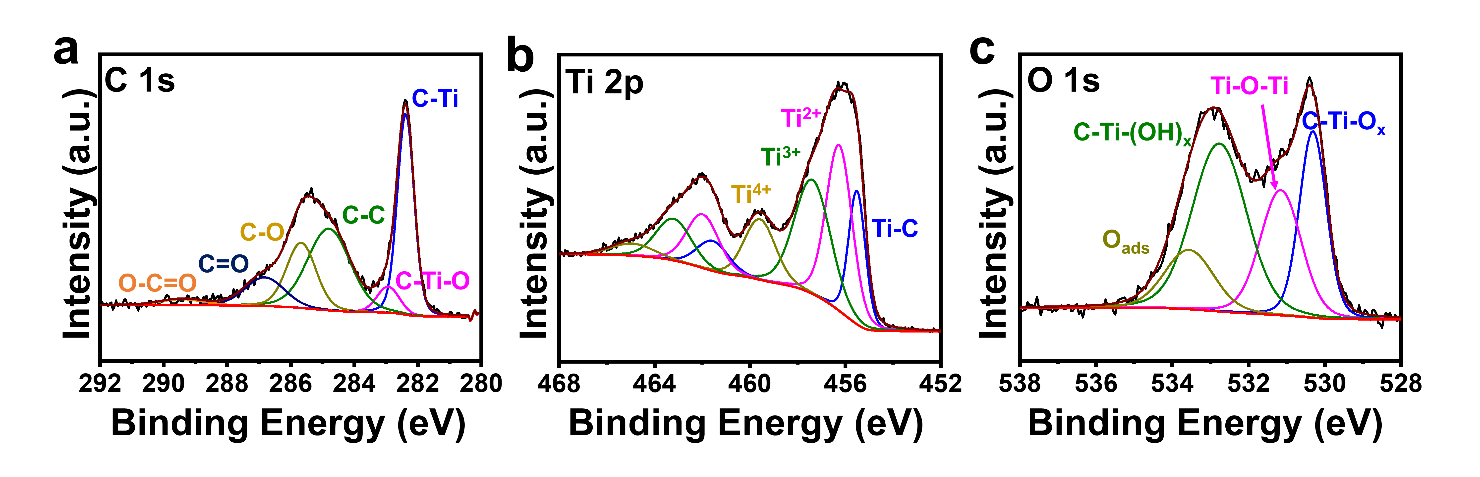


**Figure S6.** XPS spectra of AT-MXene for (a) C 1s, (b) Ti 2p, and (c) O 1s.


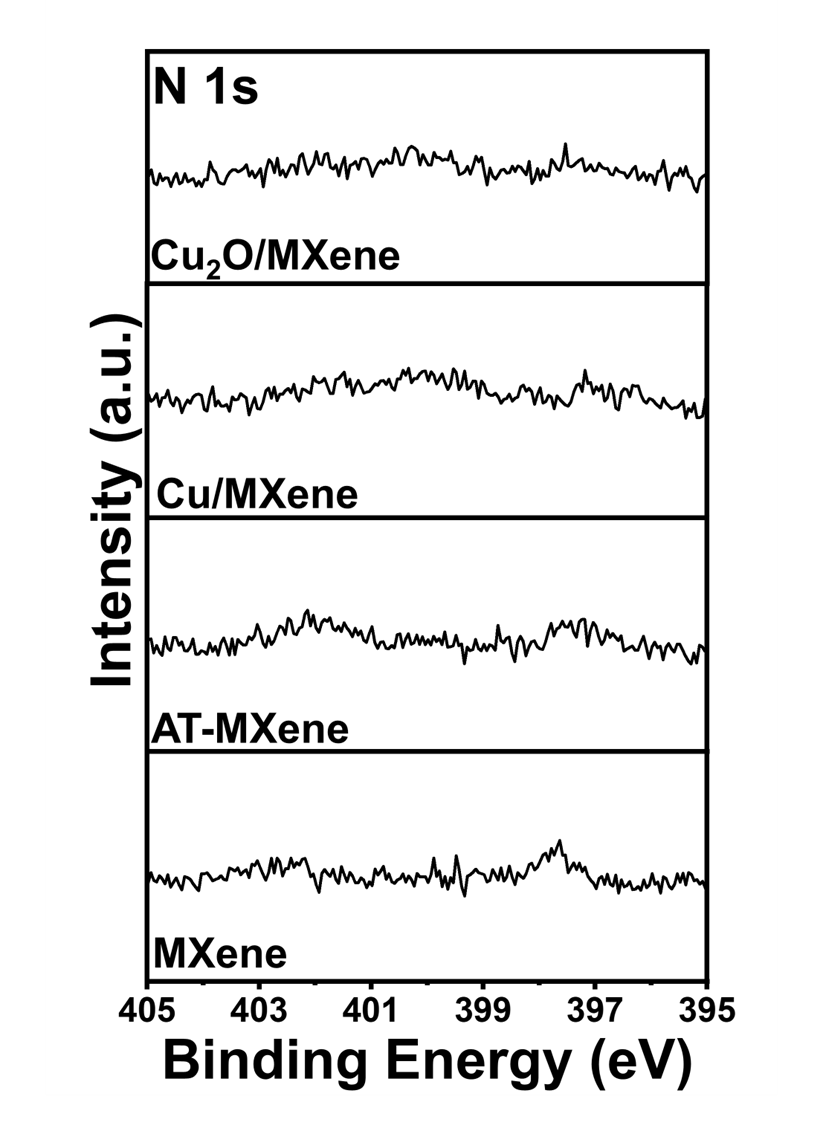


**Figure S7.** N 1s XPS spectra of Cu_2_O/MXene, Cu/MXene, AT-MXene and MXene.


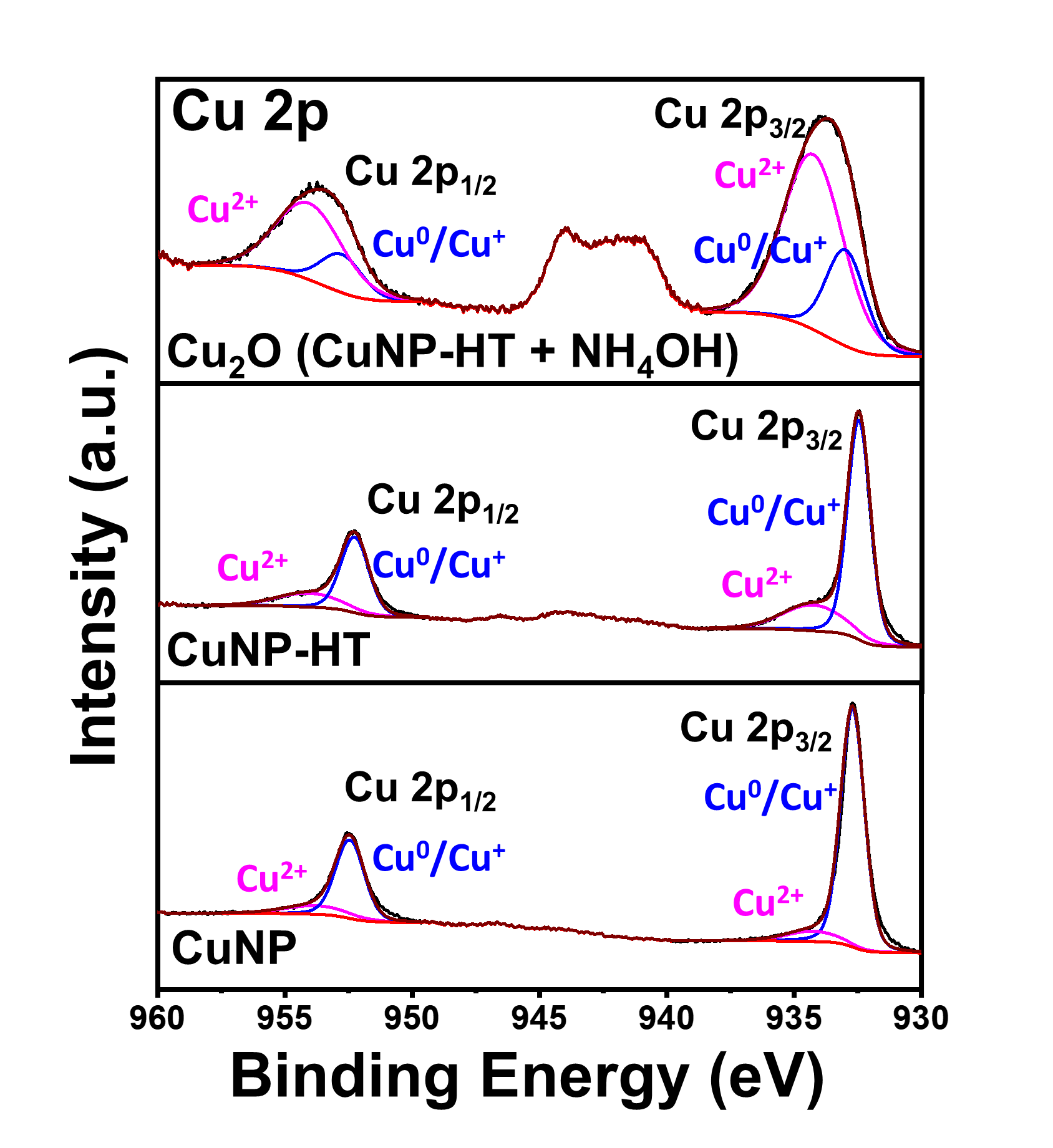


**Figure S8.** Cu 2p XPS spectra of Cu nanoparticles with three different treatments after hot injection.


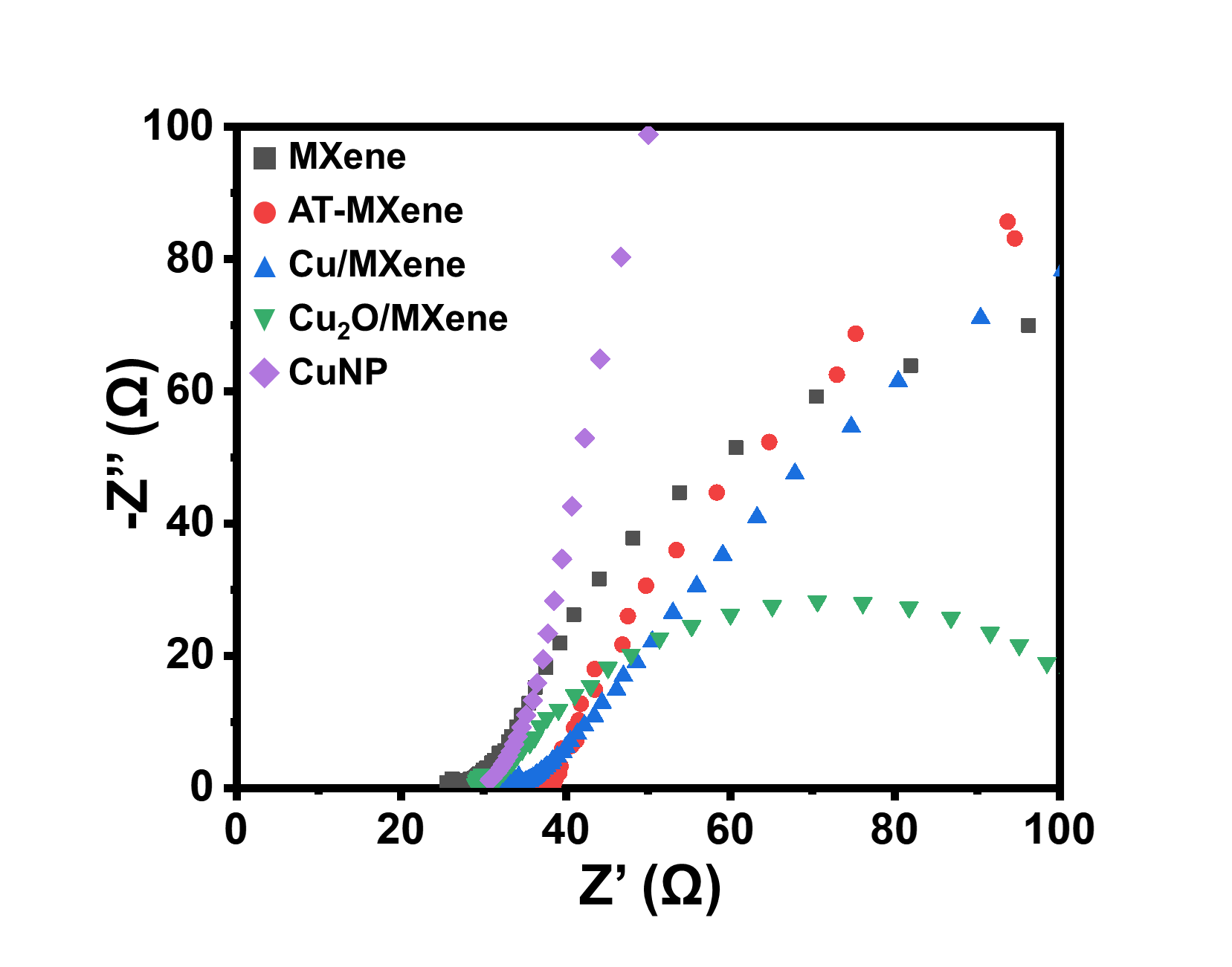


**Figure S9.** EIS of MXene, AT-MXene, Cu/MXene, Cu_2_O/MXene and CuNP in 0.1 M KHCO_3_.


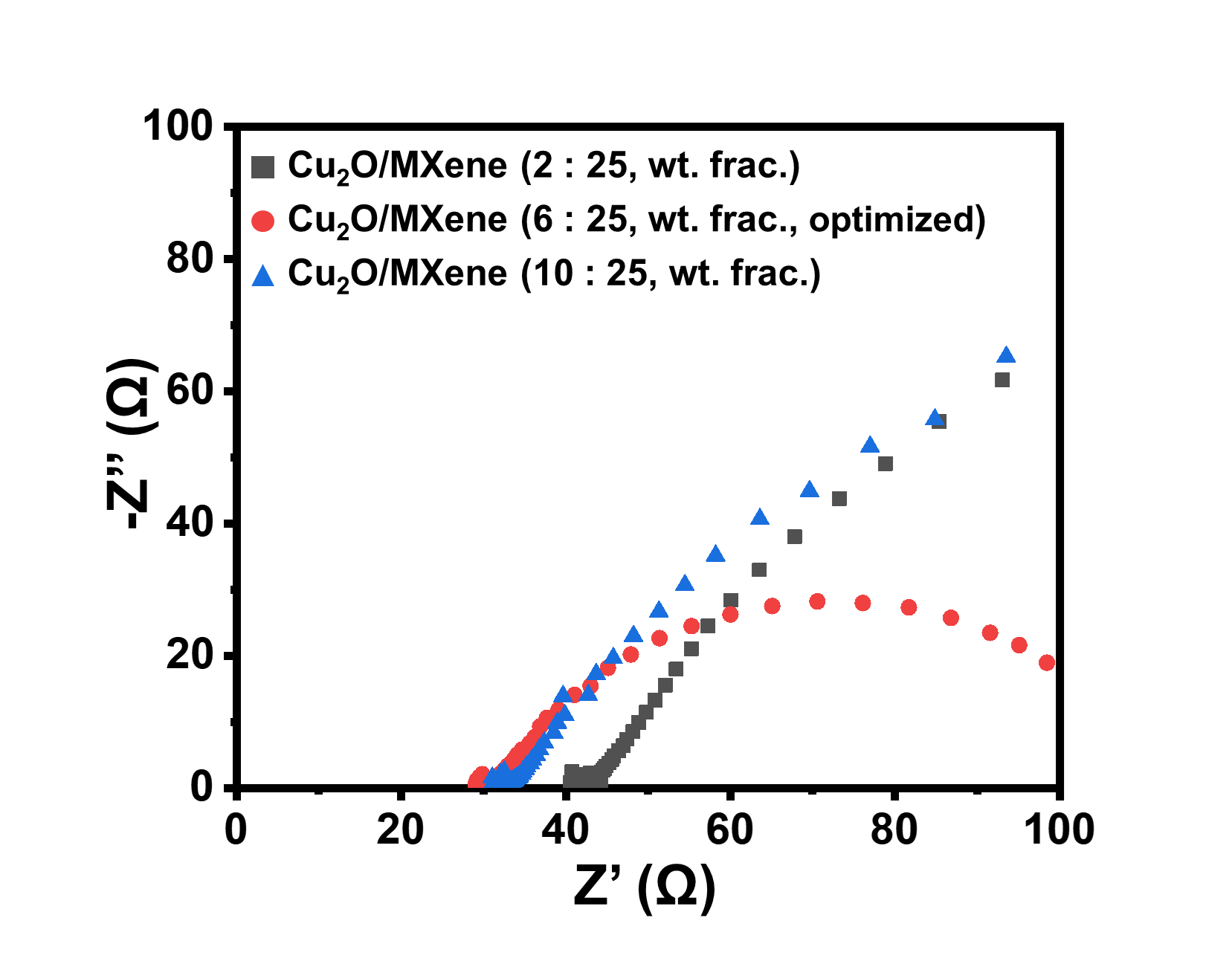


**Figure S10.** EIS of Cu_2_O/MXene with different weight ratios of CuNPs to MXene in 0.1 M KHCO_3_. Note that the red is the main sample treated in the manuscript.


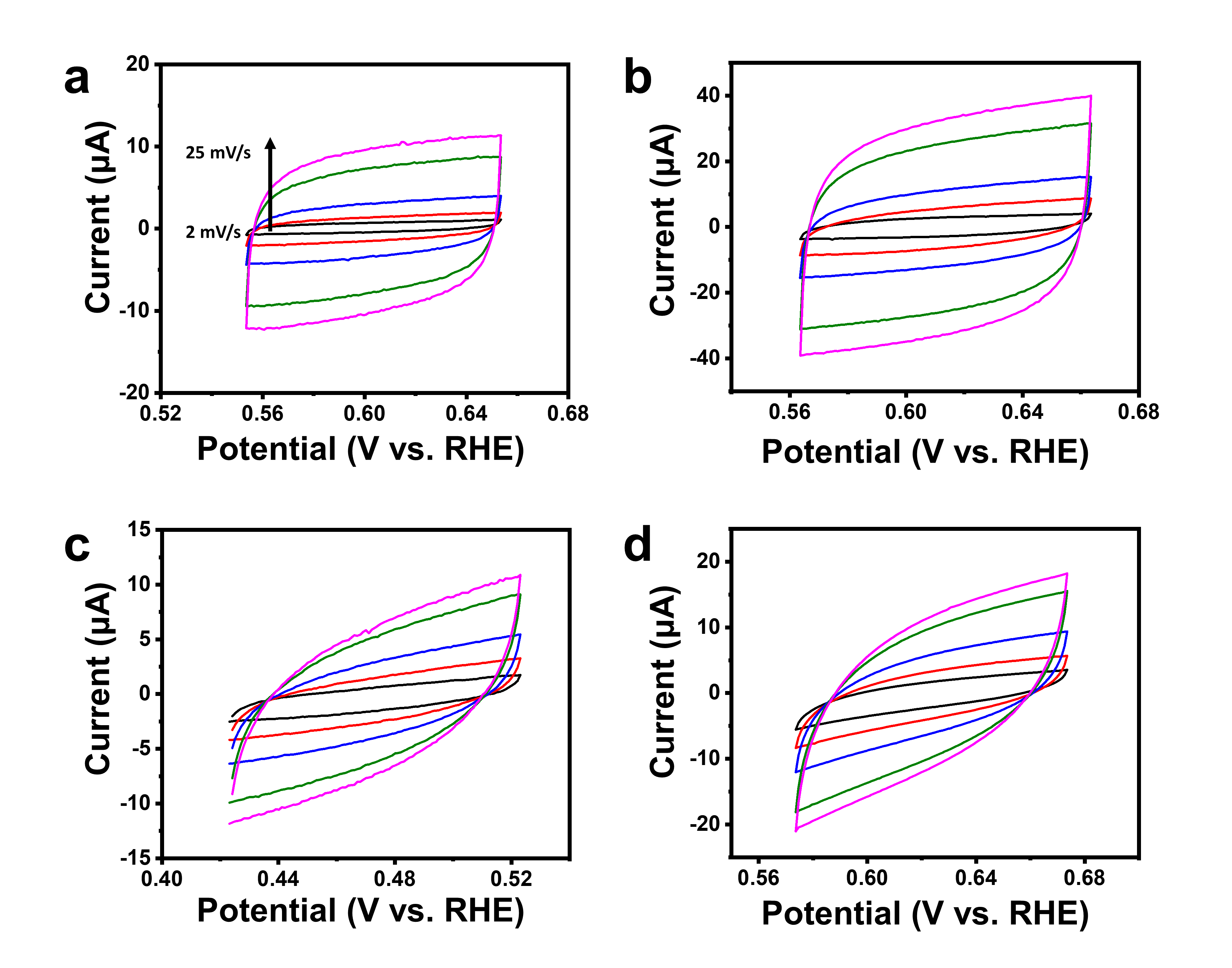


**Figure S11.** CV curves of (a) MXene, (b) AT-MXene, (c) Cu/MXene, and (d) Cu_2_O/MXene in the non-Faradaic capacitance current range, *i.e.*, centered on the open circuit potential (OCP), at scan rates of 2, 5, 10, 20, and 25 mV/s in 0.1 M KHCO_3_ for ECSA measurements. The samples were loaded on carbon cloth with 0.16 mg/cm^2^.


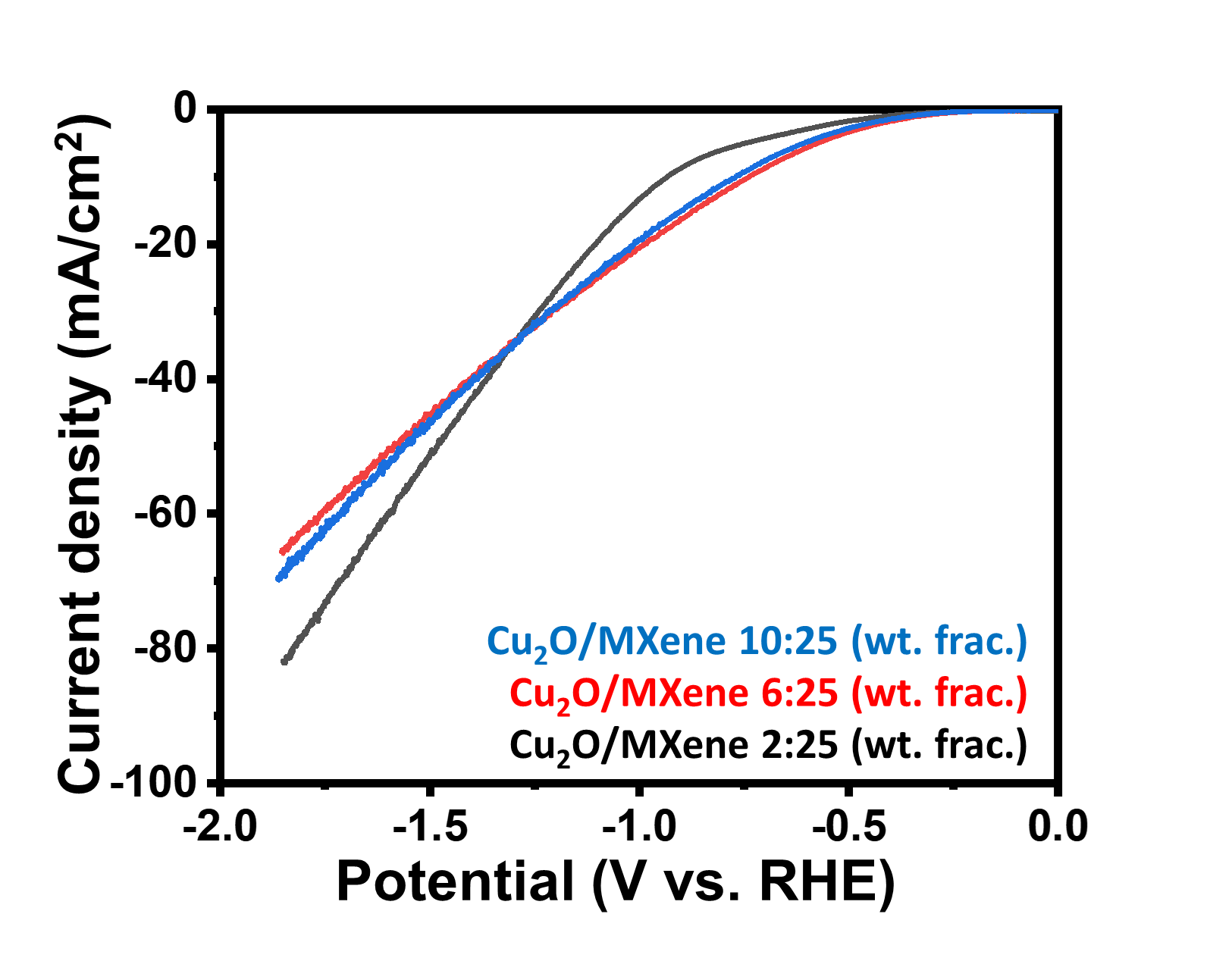


**Figure S12.** LSV curves of Cu_2_O/MXene with different Cu_2_O/MXene weight fractions in 0.1 M KHCO_3_ saturated with CO_2_.


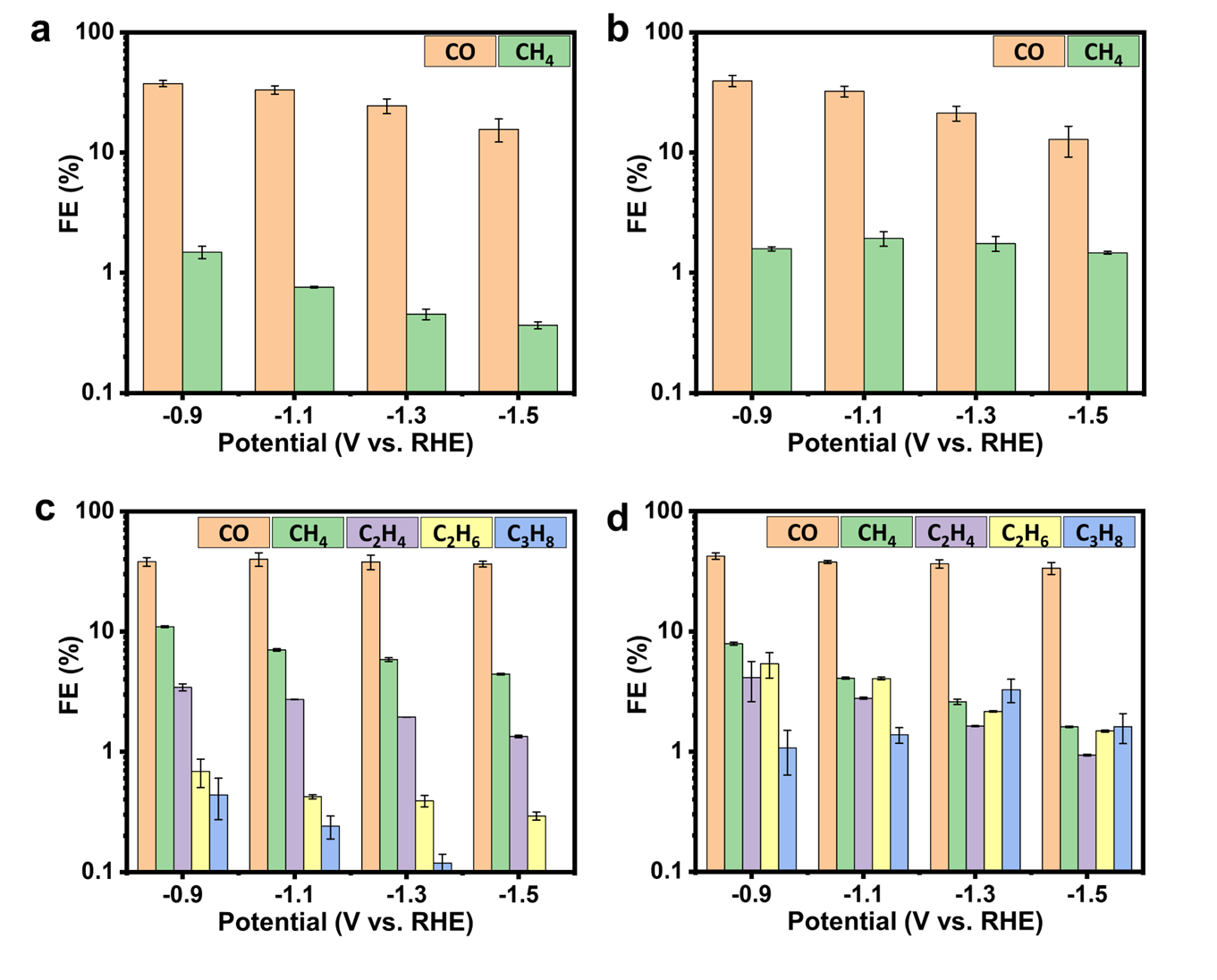


**Figure S13.** Faradaic efficiency results of (a) MXene, (b) AT-MXene, (c) Cu/MXene and (d) Cu_2_O/MXene for CO_2_ RR without H_2_ in 0.1 M KHCO_3_.


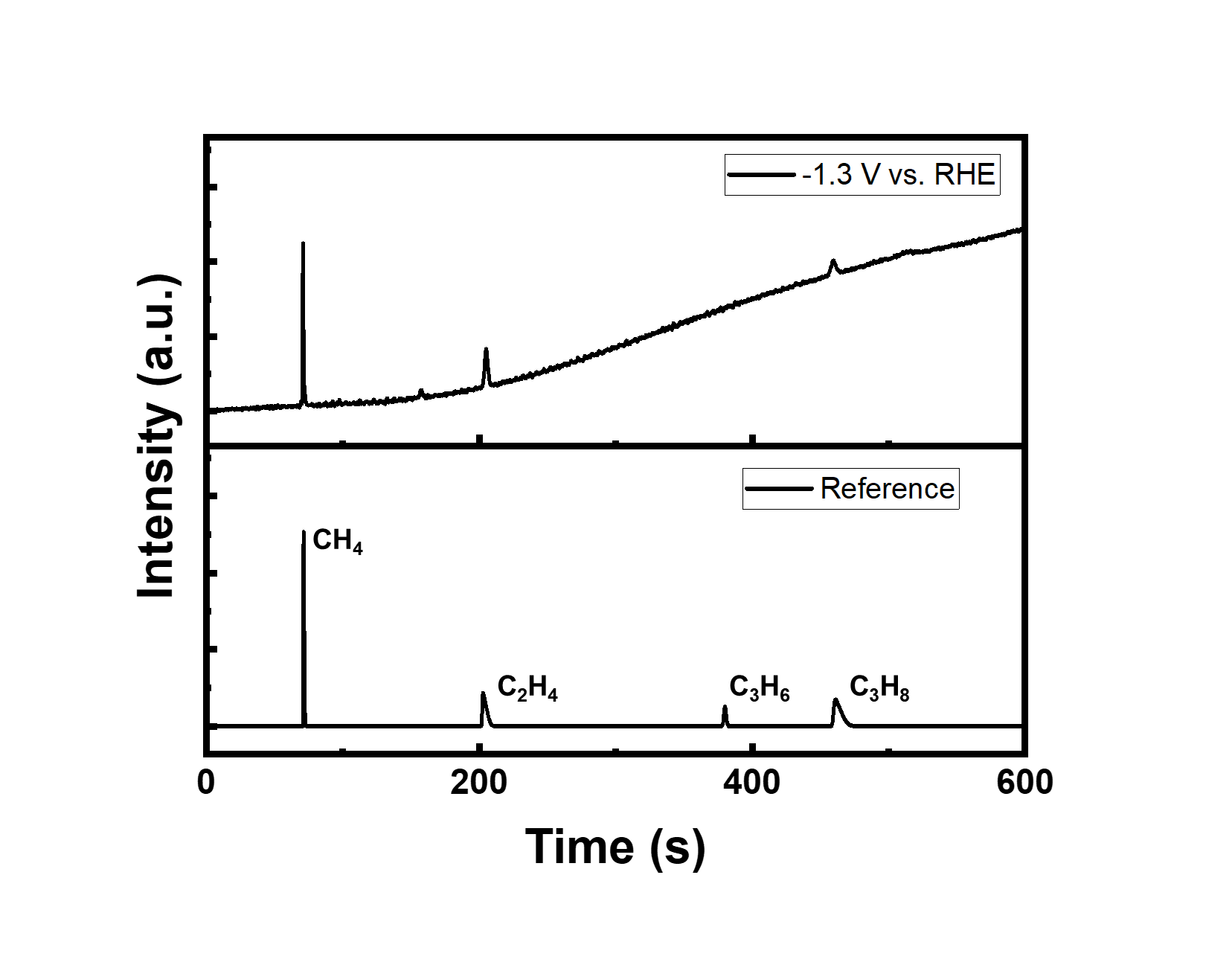


**Figure S14**. Comparison of gas chromatograms for the gaseous products from CO_2_RR on Cu_2_O/MXene at -1.3 V vs. RHE in CO_2_-saturated 0.1 M KHCO_3_ to the reference gas mixture (CH_4_, C_2_H_4_, C_3_H_6_, and C_3_H_8_).


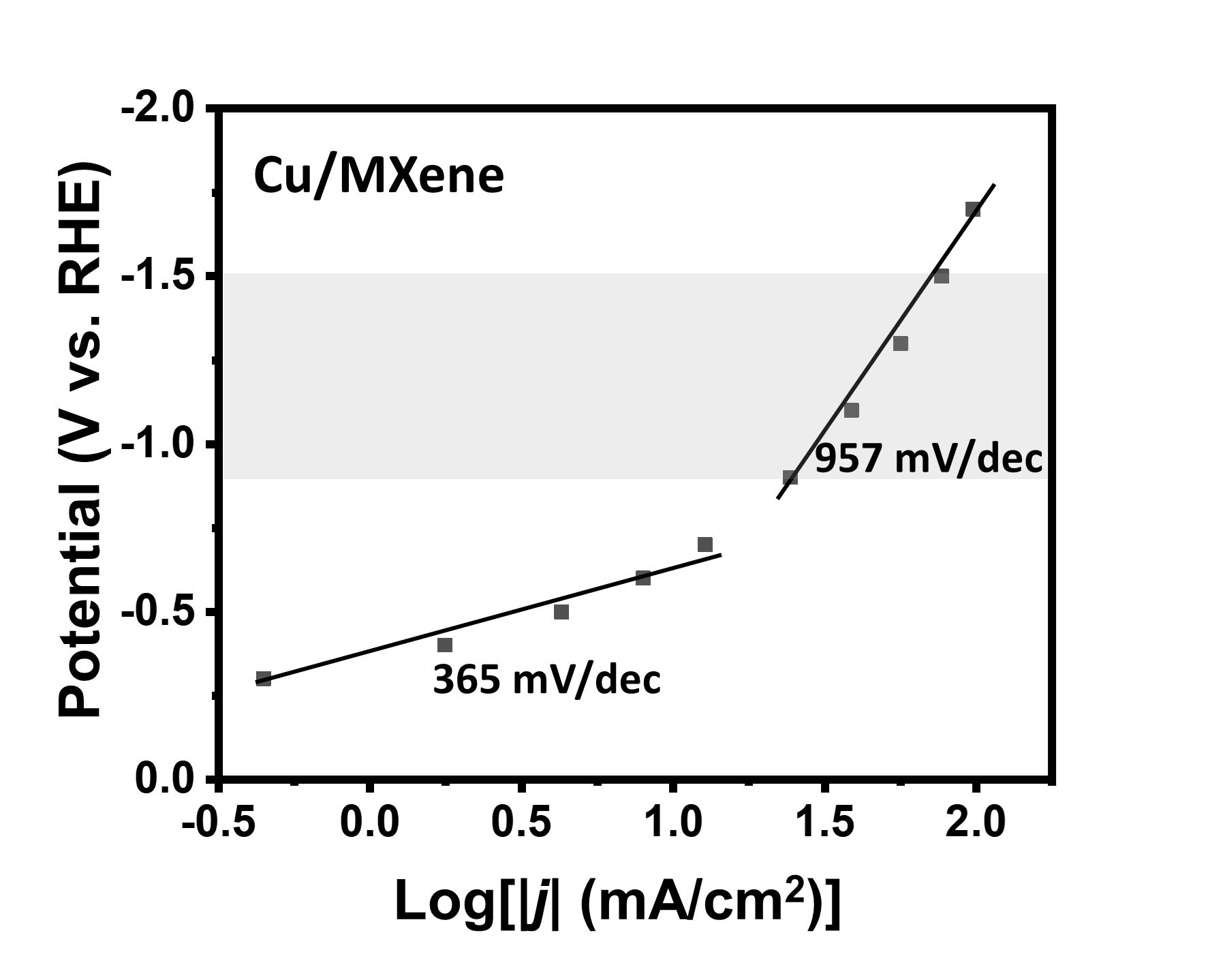


**Figure S15.** Tafel slope with Cu/MXene. The intermediate overpotential region where significant C_3_ production was detected is highlighted in grey.


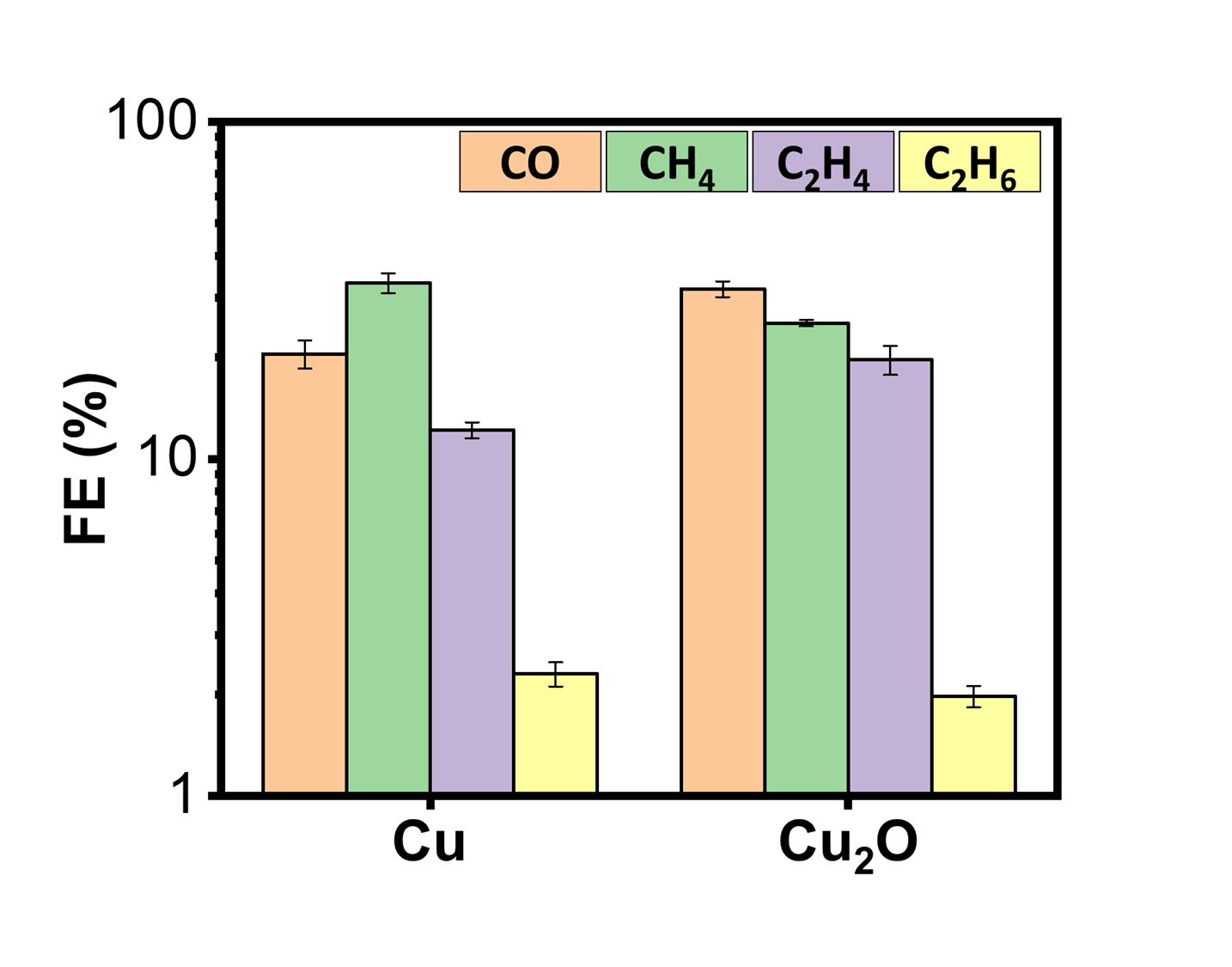


**Figure S16.** Faradaic efficiency results of Cu and Cu_2_O for CO_2_RR without H_2_ in 0.1 M KHCO_3_ at -1.3 V vs. RHE.


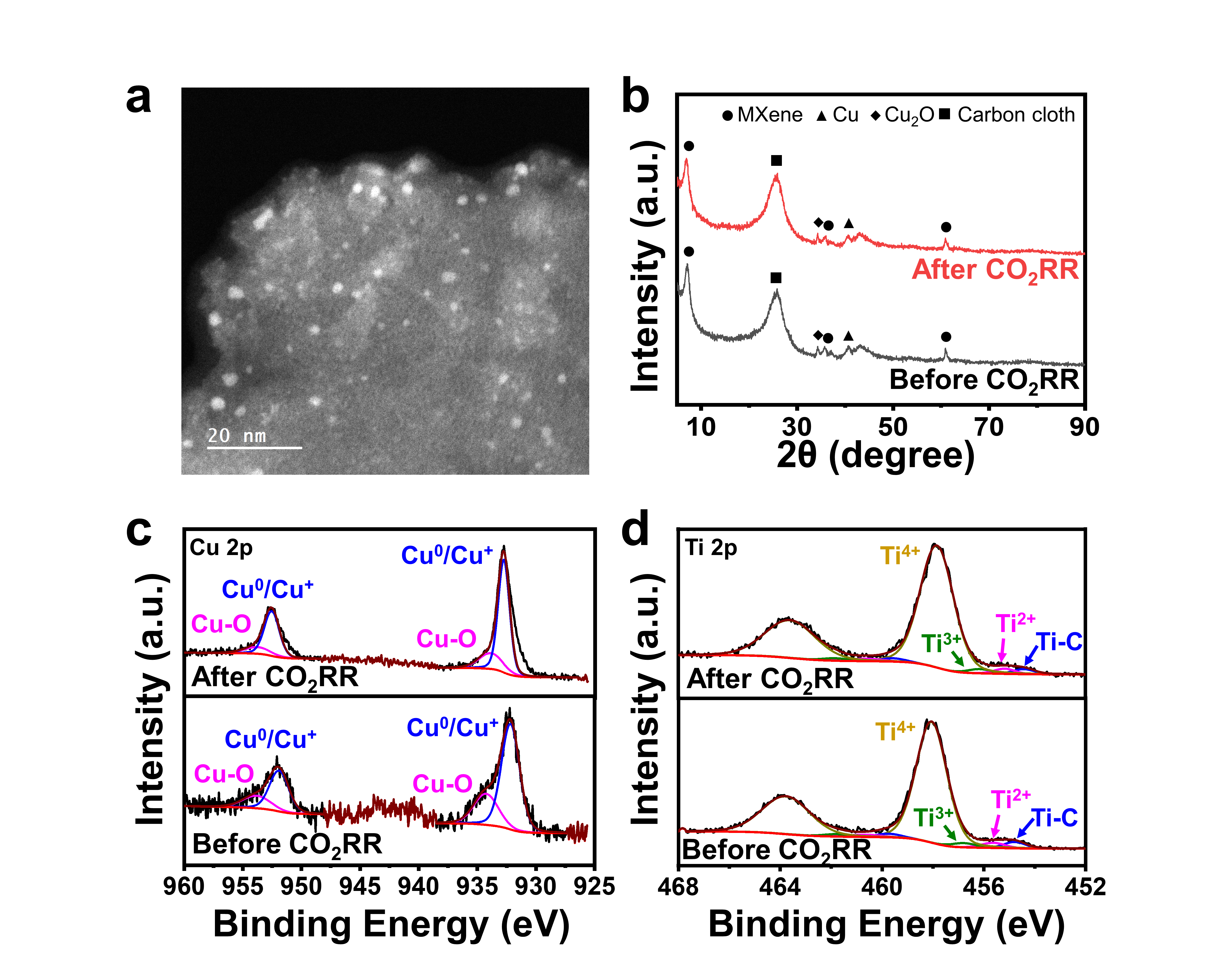


**Figure S17.** a) HAADF-STEM image, b) XRD patterns, c) Cu 2p, and d) Ti 2p XPS spectra of Cu_2_O/MXene after CO_2_RR in 0.1 M KHCO_3_.


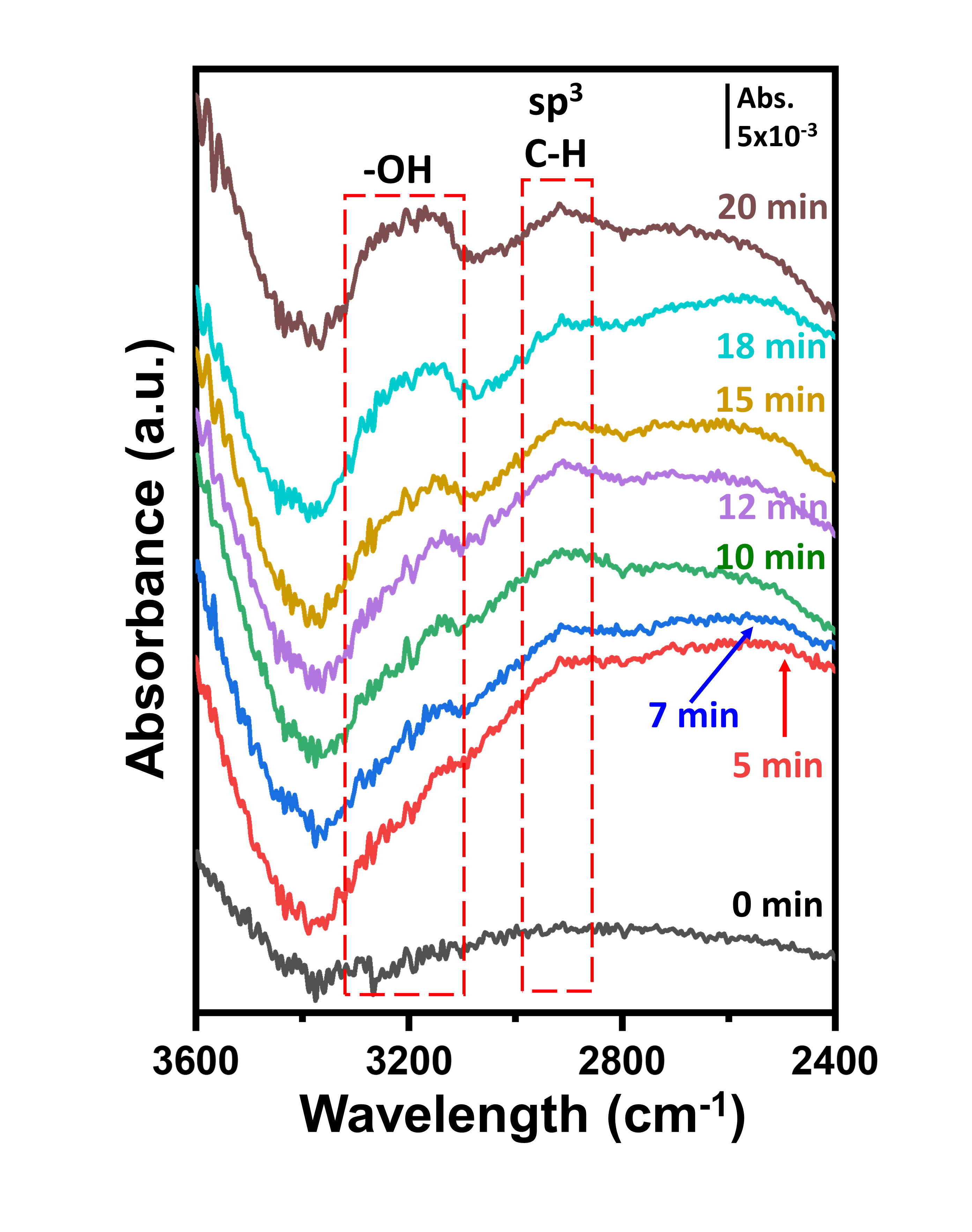
**Figure S18.** ATR-FTIR spectra for Cu_2_O/MXene during CO_2_RR, ranges of 3600–2400 cm^−1^.

The -CH_3_ asymmetric mode of the alkane structure was found in the peak range 2830–2980 cm^−1^.^[4]^ In the ranges of 3100-3340 cm^−1^, a broad -OH peak was observed, indicating that -OH adhered to the catalyst. This may result in the formation of an OH- functional group of the MXene. Specifically, the O- functional group in MXene stabilized to the OH- in an aqueous solution.^[5]^ Another reason may be proton transfer from the OH- functional group to the CO_2_ reduction intermediates and hydrogen recovery from proton-coupled electron transfer (PCET).^7,8^ Handoko et al. showed that the O-modulated MXene could accelerate PCET during CO_2_RR through experiment and DFT calculation because the O- functional group offers a site for stabilizing the *H intermediate.^[6]^ Chen et al. theoretically explained that the OH- functional group in MXene could provide an H atom to the CO_2_RR intermediate and then recover in the last step, where one final PCET donates H back to the MXene.^[7]^ Thus, these effects may affect the -OH peak in the ATR-FTIR spectrum during the CO_2_RR.

**Figure S19.** Proposed electrochemical CO_2_RR mechanism for C_3_H_8_ production on Cu_2_O/MXene
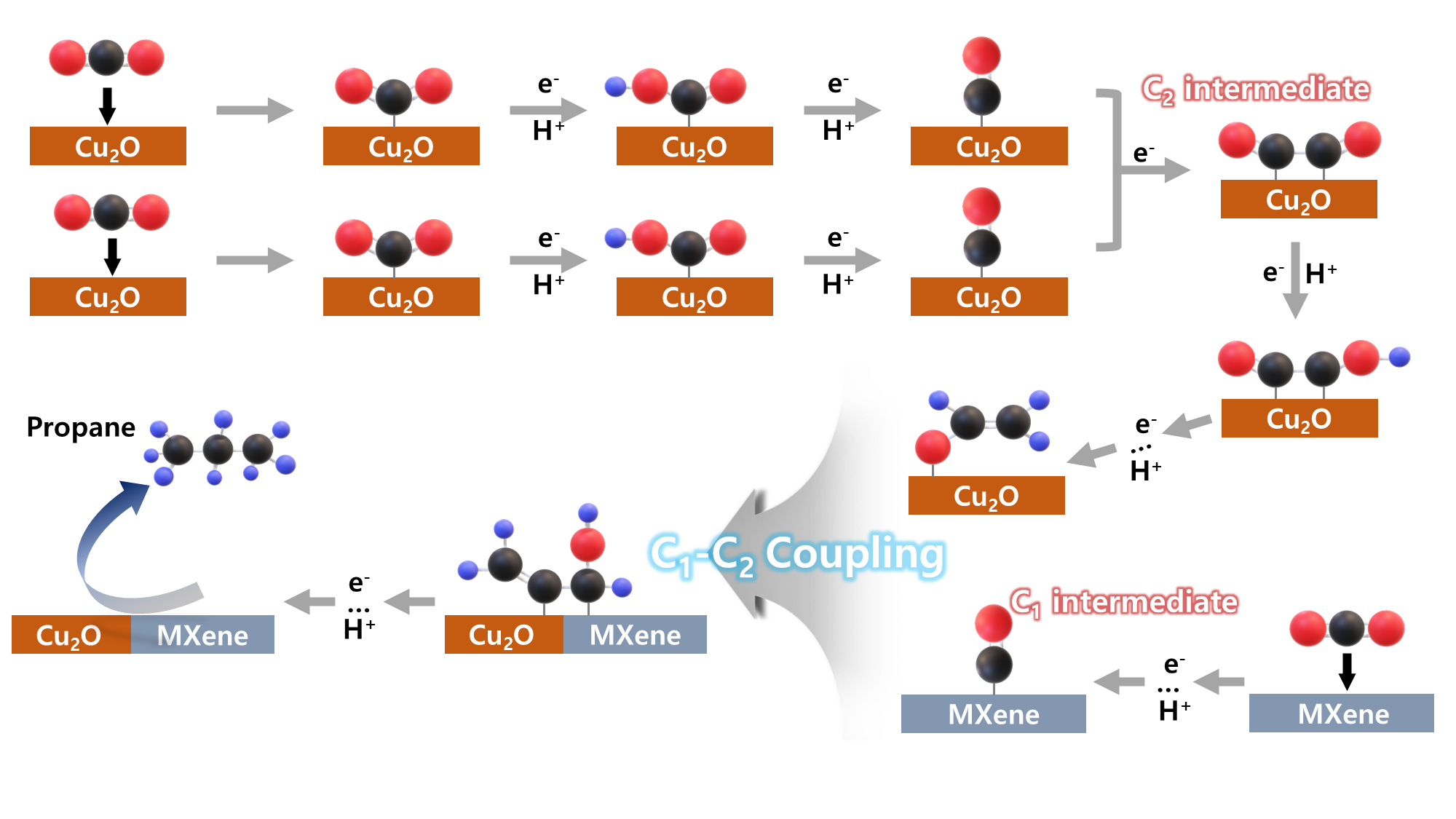
.


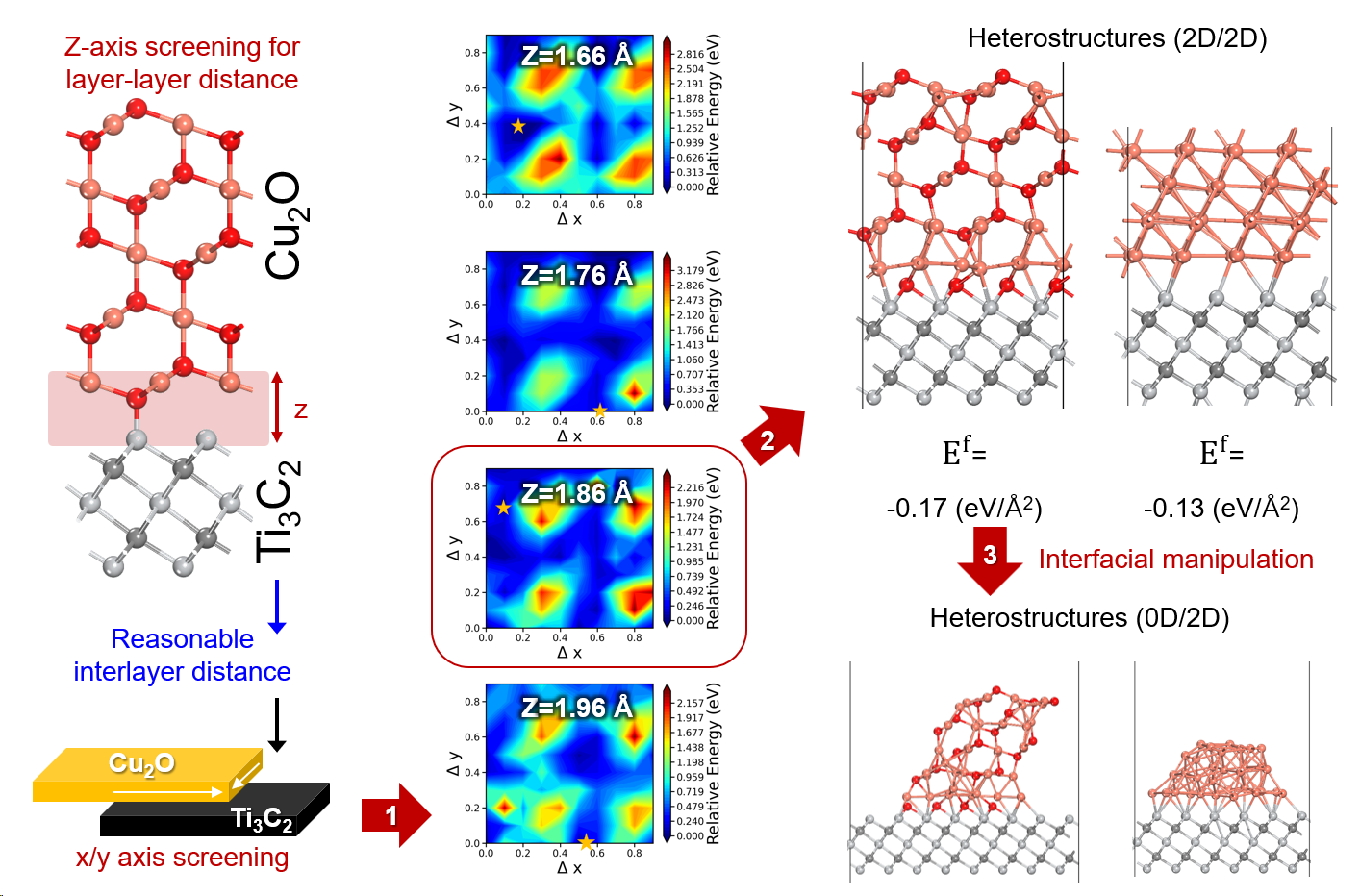


**Figure S20.** Schematic process of 2D/2D and 0D/2D heterostructure construction by scanning along the x, y, and z axis in fractional coordinates and manipulating interface to expose the MXene surface for 0D/2D heterostructure. Yellow stars represent the most stable heterostructure among various configurations.

We rationally designed the optimal 2D/2D heterostructures considering the lattice mismatch between the Cu_2_O (or Cu) and MXene and then evaluated thermodynamical stability of 2D/2D heterostructures by calculating the heterostructure formation energy ($E^{f}$), which shows energetically favorable negative values, as shown in Table S8.


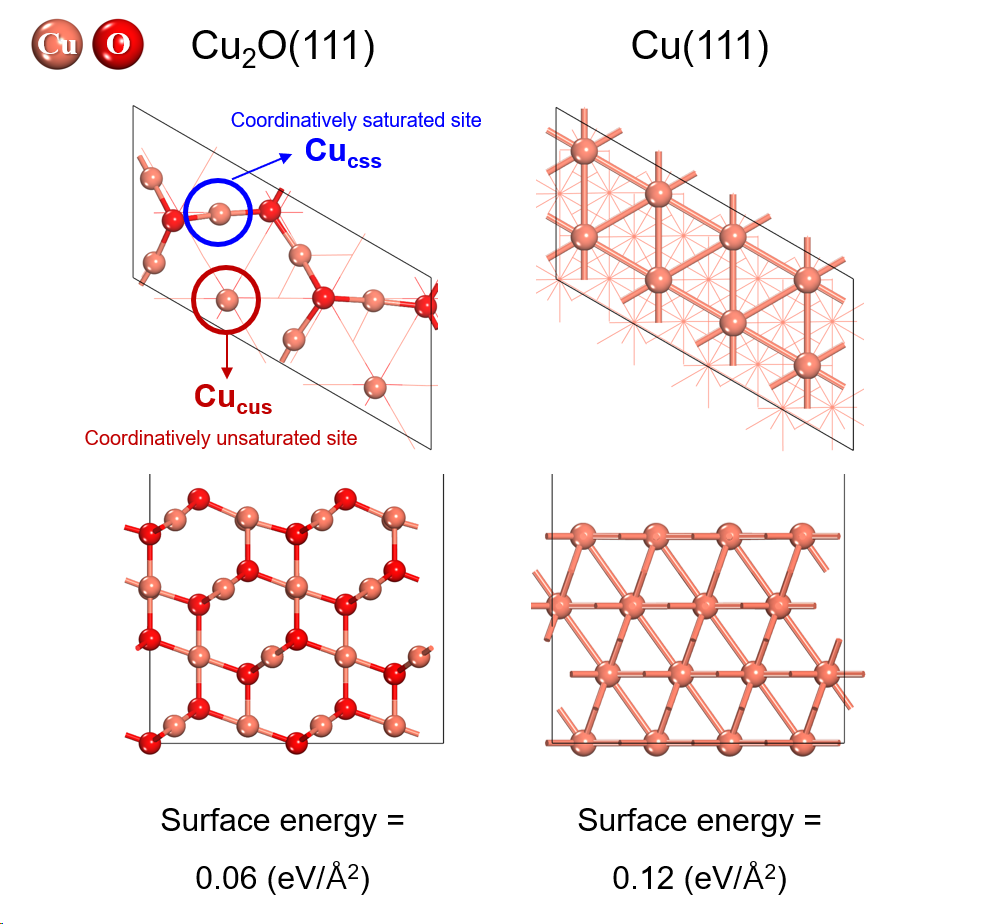


**Figure S21.** The top and side views of the pure Cu_2_O(111) and Cu(111) surface structures with their surface energy.


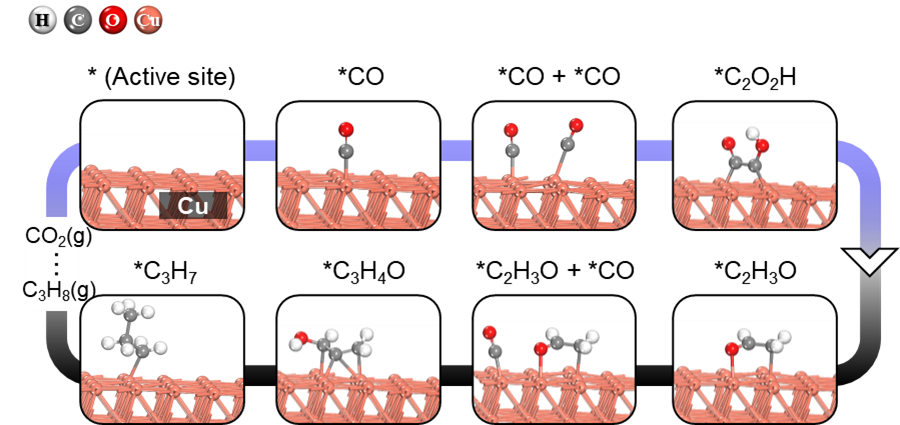


**Figure S22.** CO_2_RR mechanism and calculated free energy diagram (FED) for C_3_H_8_(g) production in pure Cu(111) structure.


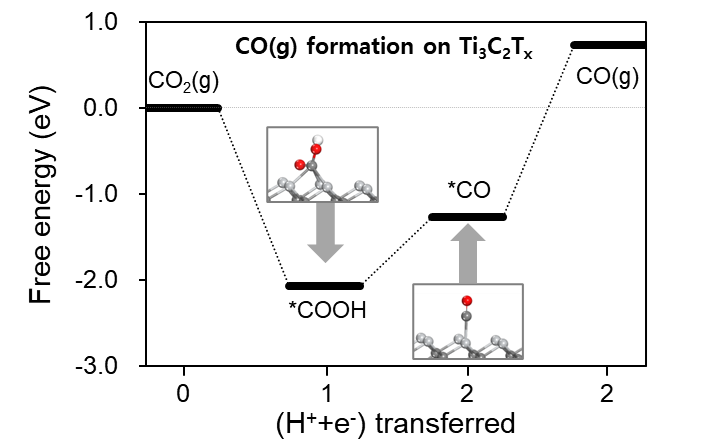


**Figure S23.** Calculated CO_2_RR free energy diagram (FED) for CO(g) production in bare MXene (Ti_3_C_2_) structure.


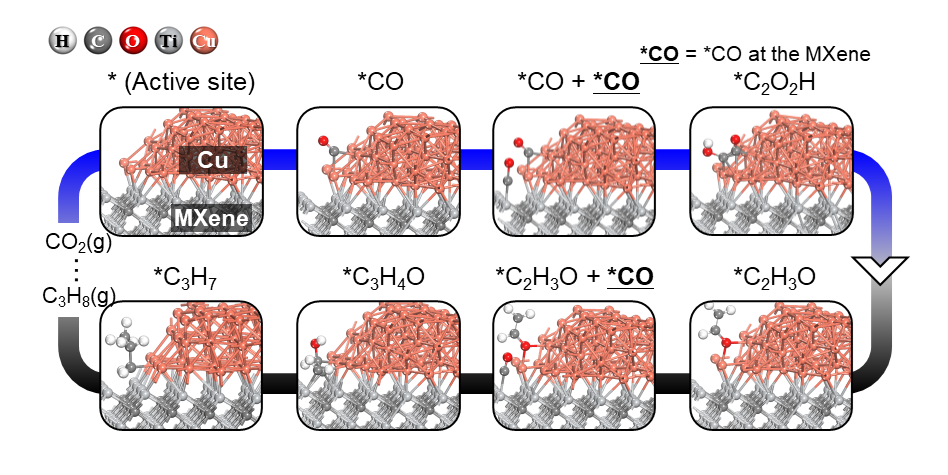


**Figure S24.** CO_2_RR mechanism and calculated free energy diagram (FED) for C_3_H_8_(g) production in 0D/2D heterostructure of Cu/MXene.

**Table S1.** Peak areas and area ratios of Ti 2p for as-prepared electrocatalysts.

| **Catalyst** | **Chemical state of Ti** | **Peak area of**  **2p 3/2** | **Peak area of**  **2p 1/2** | **Area ratio**  **(2p 3/2 over 2p 1/2)** |
| --- | --- | --- | --- | --- |
| MXene | Ti-C | 9.09 | 4.17 | ~4:2 |
|  | Ti^2+^ | 32.03 | 12.56 | ~4:2 |
|  | Ti^3+^ | 21.82 | 8.36 | ~4:2 |
|  | Ti^4+^ | 9.14 | 3.84 | ~4:2 |
| Cu/MXene | Ti-C | 9.62 | 4.95 | ~4:2 |
|  | Ti^2+^ | 8.7 | 4.2 | ~4:2 |
|  | Ti^3+^ | 4.41 | 2.3 | ~4:2 |
|  | Ti^4+^ | 46.31 | 19.77 | ~4:2 |
| Cu_2_O/MXene | Ti-C | 4.8 | 1.68 | ~4:2 |
|  | Ti^2+^ | 5.74 | 2.65 | ~4:2 |
|  | Ti^3+^ | 7.41 | 4.02 | ~4:2 |
|  | Ti^4+^ | 51.64 | 22.07 | ~4:2 |

**Table S2.** Faradaic efficiencies of product distribution at different applied potentials using different electrocatalysts.

| **Catalyst** | **Potential**  **(V vs. RHE)** | **H_2_**  **(FE, %)** | **CO**  **(FE, %)** | **CH_4_**  **(FE, %)** | **C_2_H_4_**  **(FE, %)** | **C_2_H_6_**  **(FE, %)** | **C_3_H_8_**  **(FE, %)** | **CH_3_CHO**  **(FE, %)** | **CH_3_COOH**  **(FE, %)** | **n-PrOH (FE, %)** | **C_3_H_4_O**  **(FE, %)** |
| --- | --- | --- | --- | --- | --- | --- | --- | --- | --- | --- | --- |
| Cu | -1.3 | 19.7 | 20.5 | 33.3 | 12.2 | 2.3 | - | - | - | - | - |
| Cu_2_O | -1.3 | 11.3 | 31.9 | 25.3 | 19.7 | 2.0 | - | - | - | - | - |
| MXene | -0.9 | 59.9 | 37.5 | 1.5 | - | - | - | - | - | - | - |
|  | -1.1 | 65.3 | 33.2 | 0.8 | - | - | - | - | - | - | - |
|  | -1.3 | 73.9 | 24.5 | 0.5 | - | - | - | - | - | - | - |
|  | -1.5 | 82.4 | 15.6 | 0.4 | - | - | - | - | - | - | - |
| AT-MXene | -0.9 | 56.1 | 39.5 | 1.6 | - | - | - | - | - | - | - |
|  | -1.1 | 63.6 | 32.3 | 1.9 | - | - | - | - | - | - | - |
|  | -1.3 | 74.6 | 21.2 | 1.7 | - | - | - | - | - | - | - |
|  | -1.5 | 83.5 | 12.9 | 1.5 | - | - | - | - | - | - | - |
| Cu/MXene | -0.9 | 44.3 | 38.1 | 11.0 | 3.4 | 0.7 | 0.4 | - | - | trace | - |
|  | -1.1 | 47.9 | 40.0 | 7.0 | 2.7 | 0.4 | 0.2 | - | - | trace | - |
|  | -1.3 | 52.2 | 37.9 | 5.8 | 1.9 | 0.4 | 0.1 | trace | trace | trace | - |
|  | -1.5 | 55.6 | 36.4 | 4.4 | 1.3 | 0.3 | 0.1 | trace | trace | trace | - |
| Cu_2_O/MXene | -0.9 | 36.1 | 42.4 | 7.9 | 4.1 | 5.4 | 1.1 | trace | trace | trace | trace |
|  | -1.1 | 47.4 | 37.9 | 4.1 | 2.8 | 4.1 | 1.4 | trace | trace | trace | trace |
|  | -1.3 | 52.2 | 36.6 | 2.6 | 1.6 | 2.2 | 3.3 | trace | trace | trace | trace |
|  | -1.5 | 58.2 | 33.6 | 1.6 | 0.9 | 1.5 | 1.6 | - | - | trace | trace |

**Table S3.** Comparison of electroreduction products from C_1_ feedstocks (CO_2_ and CO) using Ti_3_C_2_T_x_ MXene-based catalysts.

| **Catalyst** | **Electrolyte** | **Feedstock** | **Potential**  **(V vs. RHE)** | **H_2_**  **(FE, %)** | **CO**  **(FE, %)** | **HCOOH**  **(FE, %)** | **CH_3_OH**  **(FE, %)** | **CH_3_COOH**  **(FE, %)** | **C_2_H_5_OH**  **(FE, %)** | **CH_4_**  **(FE, %)** | **C_2_H_4_**  **(FE, %)** | **C_2_H_6_**  **(FE, %)** | **n-PrOH**  **(FE, %)** | **C_3_H_8_**  **(FE, %)** |
| --- | --- | --- | --- | --- | --- | --- | --- | --- | --- | --- | --- | --- | --- | --- |
| Cu-SA/MXene^[8]^ | 1M KOH | CO | -0.7 | 2.1 | - | - | - | 1.8 | 25.4 | - | 70.4 | - | - | - |
| Cu-NP/Ti_3_C_2_Tx^[8]^ | 1M KOH | CO | -0.7 | 38.6 | - | - | - | 8.5 | 22.2 | - | 28.6 | - | - | - |
| Ti_3_C_2_T_x_^[8]^ | 1M KOH | CO | -0.7 | 87.3 | - | - | - | - | - | - | 6.1 | 6.5 | - | - |
| Cu/Ti_3_C_2_T_x_^[9]^ | 0.1M NaHCO_3_ | CO_2_ | -1.5 (Ag/AgCl) | 20 | 3.5 | 54.7 | 19.9 | - | - | 12.1 | - | - | - | - |
| Ti_3_C_2_T_x_^[9]^ | 0.1M NaHCO_3_ | CO_2_ | -1.5 (Ag/AgCl) | 34.6 | 2 | - | - | - | - | 3.5 | - | - | - | - |
| Cu/MXene  [This work] | 0.1M KHCO_3_ | CO_2_ | -1.3 | 52.2 | 37.9 | - | - | trace | - | 5.8 | 1.9 | 0.4 | trace | 0.1 |
| Cu_2_O/MXene  [This work] | 0.1M KHCO_3_ | CO_2_ | -1.3 | 52.2 | 36.6 | - | - | trace | - | 2.6 | 1.6 | 2.2 | trace | 3.3 |

**Table S4.** Current research status of FE_C3H8_ for electrocatalytic CO_2_RR.

| **Catalysts** | **Electrolyte** | **Potential**  **(V vs. RHE)** | **FE_C3H8_**  **(%)** | **Additives** | **Ref.** |
| --- | --- | --- | --- | --- | --- |
| Cu-2 GDE | 3.5 M KOH | - | trace | - | [10] |
| Cu-Ni nanoparticle | 0.05 M KHCO_3_ | -1.2 | 0.2 | - | [11] |
| Nickel thiolate 3 | 0.1 M KHCO_3_ | -1.0 | 0.4 | - | [12] |
| Nickel thiolate 4 | 0.1 M KHCO_3_ | -1.0 | 0.4 | - | [12] |
| Nickel thiolate 5 | 0.5 M KHCO_3_ | -1.0 | 0.5 | - | [12] |
| Nickel thiolate 5 | 0.1 M KCl | -1.0 | 1.5 | - | [12] |
| Nickel thiolate 5 | 0.1 M potassium phosphate buffer | -1.0 | 0.1 | - | [12] |
| Nickel thiolate 5 | 0.1 M LiClO_4_ | -1.0 | 0.3 | - | [12] |
| PG-NaCu | Na_2_HPO_4_  /NaH_2_PO_4_ | -1.5 (Ag/AgCl) | trace | - | [13] |
| FePc | 0.1 M KHCO_3_ | -1.1 | 0.11 | - | [14] |
| FePc | 0.1 M KHCO_3_ | -1.7 | 0.21 | - | [14] |
| XCu | 0.1 M KHCO_3_ | -1.65 | trace | - | [15] |
| XFe | 0.1 M KHCO_3_ | -1.65 | trace | - | [15] |
| CuBi#50 | 0.1 M KHCO_3_ | -1.8 | 85.4 | Bidentate carbonate | [16] |
| ImF-Mo_3_P | 1 M KOH | -0.8 | 91 | 1-ethyle-3-methyle imidazolium | [17] |

**Table S5.** TOF of CO_2_RR products for Cu/MXene and Cu_2_O/MXene in 0.1 M KHCO_3_ at −1.3 V vs. RHE.

|  |  | **TOF (10^-3^**$\boldsymbol{\times}$**1/h)** | | | |
| --- | --- | --- | --- | --- | --- |
| **Catalyst** | **Loading amount** | **CH_4_** | **C_2_H_4_** | **C_2_H_6_** | **C_3_H_8_** |
| MXene | 4.37 mmol | 2.98 | - | - | - |
| AT-MXene | 4.37 mmol | 8.27 | - | - | - |
| Cu/MXene | 3.70 mmol | 37.45 | 17.09 | 2.87 | 1.26 |
| Cu_2_O/MXene | 3.70 mmol | 15.24 | 11.78 | 15.36 | 15.96 |

**Table S6.** Calculated thermodynamic values of ZPE and TDS (298K) for all species including gas molecules and reaction intermediates at the active site of catalysts during CO_2_RR.

| **Species** | **ZPE (eV)** | **TDS (eV)** |
| --- | --- | --- |
| CO_2_(g) | 0.31 | 0.65 |
| H_2_O(g) | 0.56 | 0.27 |
| H_2_(g) | 0.67 | 0.41 |
| CO(g) | 0.14 | 0.67 |
| C_3_H_8_(g) | 2.79 | 0.47 |
| *COOH | 0.63 | 0.19 |
| *CO | 0.17 | 0.21 |
| *CO+*CO | 0.42 | 0.25 |
| *C_2_O_2_H | 0.74 | 0.23 |
| *C_2_H_3_O | 1.21 | 0.28 |
| *C_2_H_3_O+*CO | 1.38 | 0.46 |
| *C_3_H_4_O | 1.21 | 0.28 |
| *C_3_H_7_ | 2.37 | 0.28 |

**Table S7.** Calculated adsorption Gibbs free energies ($\Delta G_{\mathrm{ads}^{*}}$) for CO_2_RR in heterostructures of Cu_2_O/MXene and Cu/MXene and pure Cu_2_O(111) and Cu(111) surfaces according to the transferred (H^+^+e^-^).

| **(H^+^+e^-^) transferred** | **Reaction step** | **DG_ads*_ (eV)** | | | |
| --- | --- | --- | --- | --- | --- |
|  |  | **Cu_2_O(111)** | **Cu(111)** | **Cu_2_O/MXene** | **Cu/MXene** |
| 1 | *COOH | 0.14 | 0.75 | -0.41 | 0.20 |
| 2 | *CO | -1.28 | -0.38 | 0.13 | -0.71 |
| 4 | *CO+*CO | 0.17 | 0.28 | -0.01 | 0.10 |
| 5 | *C_2_O_2_H | 0.79 | 1.48 | 0.80 | 0.92 |
| 9 | *C_2_H_3_O | -0.52 | -0.32 | 0.19 | -0.69 |
| 11 | *C_2_H_3_O+*CO | -1.08 | -0.25 | -0.49 | -1.22 |
| 14 | *C_3_H_4_O | -1.17 | -0.30 | -0.59 | -2.04 |
| 19 | *C_3_H_7_ | -2.21 | -1.48 | -2.01 | -3.78 |
| 20 | C_3_H_8_(g) | -1.80 | -1.80 | -1.80 | -1.80 |

**Table S8.** Lattice parameters of pure Cu_2_O(111) and Cu(111) surfaces with respect to compressive or tensile strain compared to MXene structures including heterostructure formation energy ($E^{f}$).

| **Structure** | **Lattice parameter (Å)** | **Strain (%)** | $\mathbf{E}^{\mathbf{f}}$ **(eV/Å^2^)** |
| --- | --- | --- | --- |
| Cu_2_O(111) | 12.80 | -4.57 | -0.17 |
| Cu(111) | 10.68 | +14.40 | -0.13 |
| Ti_3_C_2_ | 12.22 | - | - |

**Table S9.** Calculated adsorption Gibbs free energies ($\Delta G_{\mathrm{ads}^{*}}$) for CO_2_RR of bare MXene (Ti_3_C_2_) for CO(g) formation according to the transferred (H^+^+e^-^).

| **(H^+^+e^-^) transferred** | **Reaction step** | **DG_ads*_ (eV)** |
| --- | --- | --- |
| 1 | *COOH | -2.07 |
| 2 | *CO | -1.27 |
| 2 | CO(g) | 0.73 |

**Table S10.** Calculated reaction Gibbs free energy (DG) for *C_2_ (*C_2_O_2_H) and *C_3_ (*C_3_H_4_O) formation by sequential processes of additional *CO supply/C-C coupling for CO_2_RR in heterostructures of Cu_2_O/MXene and Cu/MXene and pure Cu_2_O(111) and Cu(111) surfaces (DG for *C_2_/*C_3_ formation = DG for *CO supply + DG for C-C coupling).

| **Structure** | **DG for CO* supply (eV)** | | **DG for C-C coupling (eV)** | | **DG for C_2_*/C_3_* formation (eV)** | |
| --- | --- | --- | --- | --- | --- | --- |
|  | *CO 🡪 *CO+*CO | *C_2_H_3_O 🡪 *C_2_H_3_O+*CO | *CO+*CO 🡪 *C_2_O_2_H | *C_2_H_3_O+*CO 🡪 *C_3_H_4_O | *CO 🡪  *C_2_O_2_H | *C_2_H_3_O 🡪 *C_3_H_4_O |
| Cu_2_O  (111) | 1.45 | -0.55 | 0.62 | -0.09 | 2.07 | -0.65 |
| Cu_2_O  /MXene | -0.14 | -0.68 | 0.81 | -0.10 | 0.67 | -0.78 |
| Cu  (111) | 0.66 | 0.07 | 1.20 | -0.05 | 1.86 | 0.02 |
| Cu  /MXene | 0.81 | -0.52 | 0.82 | -0.83 | 1.63 | -1.35 |

**Supplementary Reference**

[1] J. K. Norskov, T. Bligaard, A. Logadottir, J. R. Kitchin, J. G. Chen, S. Pandelov, J. K. Norskov, *J Electrochem Soc* **2005**, 152, J23.

[2] Y. H. Bai, B. W. J. Chen, G. W. Peng, M. Mavrikakis, *Catal Sci Technol* **2018**, 8, 3321.

[3] S. R. Kelly, C. Kirk, K. Chan, J. K. Nørskov, *J Phys Chem C* **June 11, 2020**.

[4] a) V. C. Holmberg, B. A. Korgel, *Chem Mater* **2010**, 22, 3698; b) J. Wood, M. J. Alldrick, J. M. Winterbottom, E. H. Stitt, S. Bailey, *Catal Today* **2007**, 128, 52.

[5] G. P. Gao, A. P. O'Mullane, A. J. Du, *ACS Catal* **2017**, 7, 494.

[6] A. D. Handoko, H. T. Chen, Y. W. Lum, Q. F. Zhang, B. Anasori, Z. W. Seh, *iScience* **2020**, 23, 101181.

[7] H. T. Chen, A. D. Handoko, J. W. Xiao, X. Feng, Y. C. Fan, T. S. Wang, D. Legut, Z. W. Seh, Q. F. Zhang, *ACS Appl Mater Interfaces* **2019**, 11, 36571.

[8] H. H. Bao, Y. Qiu, X. Y. Peng, J. A. Wang, Y. Y. Mi, S. Z. Zhao, X. J. Liu, Y. F. Liu, R. Cao, L. C. Zhuo, J. Q. Ren, J. Q. Sun, J. Luo, X. P. Sun, *Nat Commun* **2021**, 12, 1.

[9] K. Eid, Q. Q. Lu, S. Abdel-Azeim, A. Soliman, A. M. Abdullah, A. M. Abdelgwad, R. P. Forbes, K. I. Ozoemena, R. S. Varma, M. F. Shibl, *J Mater Chem A* **2022**, 10, 1965.

[10] H. Ren, M. Kovalev, Z. Weng, M. Z. Muhamad, H. Ma, Y. Sheng, L. Sun, J. Wang, S. Rihm, W. Yang, A. A. Lapkin, J. W. Ager, *Nat Catal* 2022, 5, 1169.

[11] T. M. Suzuki, T. Ishizaki, S. Kosaka, N. Takahashi, N. Isomura, J. Seki, Y. Matsuoka, K. Oh-Ishi, A. Oshima, K. Kitazumi, K. Sekizawa, T. Morikawa, *Chem Commun* 2020, 56, 15008

[12] J. Du, B. Cheng, H. Yuan, Y. Tao, Y. Chen, M. Ming, Z. Han, R. Eisenberg, *Angew Chem Int Ed* 2023, 62.

[13] K. V. Kholin, M. N. Khrizanforov, V. M. Babaev, G. R. Nizameeva, S. T. Minzanova, M. K. Kadirov, Y. H. Budnikova, *Molecules* 2021, 26, 5524.

[14] S.-T. Dong, C. Xu, B. Lassalle-Kaiser, *Chem Sci* 2023, 14, 550.

[15] A. F. Pérez-Cadenas, C. H. Ros, S. Morales-Torres, M. Pérez-Cadenas, P. J. Kooyman, C. Moreno-Castilla, F. Kapteijn, *Carbon* 2013, 56, 324.

[16] C. Azenha, C. Mateos-Pedrero, M. Alvarez-Guerra, A. Irabien, A. Mendes, *Chem Eng J* 2022, 445, 136575.

[17] M. Esmaeilirad, Z. Jiang, A. M. Harzandi, A. Kondori, M. Tamadoni Saray, C. U. Segre, R. Shahbazian-Yassar, A. M. Rappe, M. Asadi, *Nat Energy* **2023**, 8, 891.
